# Genomic analysis of an Argentinean isolate of *Spodoptera frugiperda granulovirus* reveals that various baculoviruses code for Lef-7 proteins with three F-box domains

**DOI:** 10.1371/journal.pone.0202598

**Published:** 2018-08-22

**Authors:** María Leticia Ferrelli, Matías Luis Pidre, Pablo Daniel Ghiringhelli, Sofía Torres, María Laura Fabre, Tomás Masson, Maia Tatiana Cédola, Alicia Sciocco-Cap, Víctor Romanowski

**Affiliations:** 1 Instituto de Biotecnología y Biología Molecular (IBBM, UNLP-CONICET), Facultad de Ciencias Exactas, Universidad Nacional de La Plata, La Plata, Buenos Aires, Argentina; 2 Laboratorio de Ingeniería Genética y Biología Celular y Molecular—Área Virosis de Insectos (LIGBCM—AVI), Departamento de Ciencia y Tecnología, Universidad Nacional de Quilmes, Bernal, Buenos Aires, Argentina; 3 IMYZA-CICVyA, Instituto Nacional de Tecnología Agropecuaria (INTA), CC 25 (B1712WAA) Castelar, Buenos Aires, Argentina; Indian Institute of Technology Delhi, INDIA

## Abstract

A new isolate of the *Spodoptera frugiperda granulovirus*, SfGV ARG, was completely sequenced and analyzed. The SfGV ARG genome is 139,812 bp long and encodes 151 putative open reading frames. Of these ORFs, 56 were found in betabaculoviruses, 19 of which are present only in GVs closely related to SfGV. Seven ORFs found homologs in this small GV group and also in noctuid NPVs. ORF066 codes a 74 amino acid protein, overlapped with *nudix* gene, with several homologs in baculovirus, found by tblastn search. Comparison with the genome of the Colombian isolate SfGV VG008 resulted in SfGV being 1101 bp smaller and lacking a homologue of VG008 ORF084, which codes for Lef-7. However, we found that ORF051 shows remote homology to Lef-7 proteins. Moreover, analysis of ORF051 along with Lef-7 proteins coded by a group of noctuid specific GVs and NPVs indicated that Lef-7 proteins coded by these viruses include three F-box domains in contrast to the single one reported for AcMNPV Lef-7. SfGV ARG genome also contains a split photolyase as a distinct feature not found in VG008. BlastX analysis revealed that a complete photolyase is coded considering a putative frameshift in a poly-A tract, which resembles known slippery sequences involved in programmed ribosome frameshifting.

## Introduction

Members of the family *Baculoviridae* are rod-shaped, insect-specific viruses with double stranded large circular DNA genomes of 80–180 kb. This family was formerly classified in two genera encompassing nucleopolyhedroviruses (NPV) and granuloviruses (GV) but more recently it was subdivided into four genera, of which *Alphabaculovirus* and *Betabaculovirus* are lepidopteran-specific NPVs and GVs, respectively [1]. Baculoviruses produce two virion forms, budded virus (BV) and occlusion-derived virus (ODV). BVs are produced at the initial stage of the cycle and are responsible for the systemic infection inside the host, whereas ODVs are produced at later stages and are specialized in the primary infection in the host midgut. ODVs are subsequently assembled into occlusion bodies (OB) consisting of a highly expressed viral protein, known as polyhedrin (Alpha-, Delta- and Gammabaculoviruses) or granulin (Betabaculoviruses) [2]. Baculoviruses have been used as targeted biocontrol agents in many countries [3] and for heterologous gene expression in insect cells by means of the baculovirus expression vector system (BEVS) [4].

The fall armyworm *Spodoptera frugiperda* (Lepidoptera: Noctuidae) is an important pest causing economic loss in several crops, mainly corn fields in the American continent, although its presence was recently reported in Africa [5, 6]. Its chemical control has become ineffective due to the emergence of resistance [7]. Therefore, the application of different control alternatives is of great concern. The use of baculoviruses as biological control agents is an interesting tool since the risk of emergence of resistance is very low.

Viruses of the *Alphabaculovirus* and *Betabaculovirus* genera have been isolated from the fall armyworm: SfMNPV and SfGV. SfGV is a slow killing virus as it takes more than a week to produce the larval death, but with the potential of enhancing the infection caused by a NPV [8]. Here we describe the genome of an Argentinean isolate of SfGV and compare it with the genome of the recently reported Colombian isolate, SfGV VG008 [9] (hereafter VG008) and other baculoviruses.

## Results and discussion

### Sequence analysis of SfGV ARG genome

**Fig 1:**
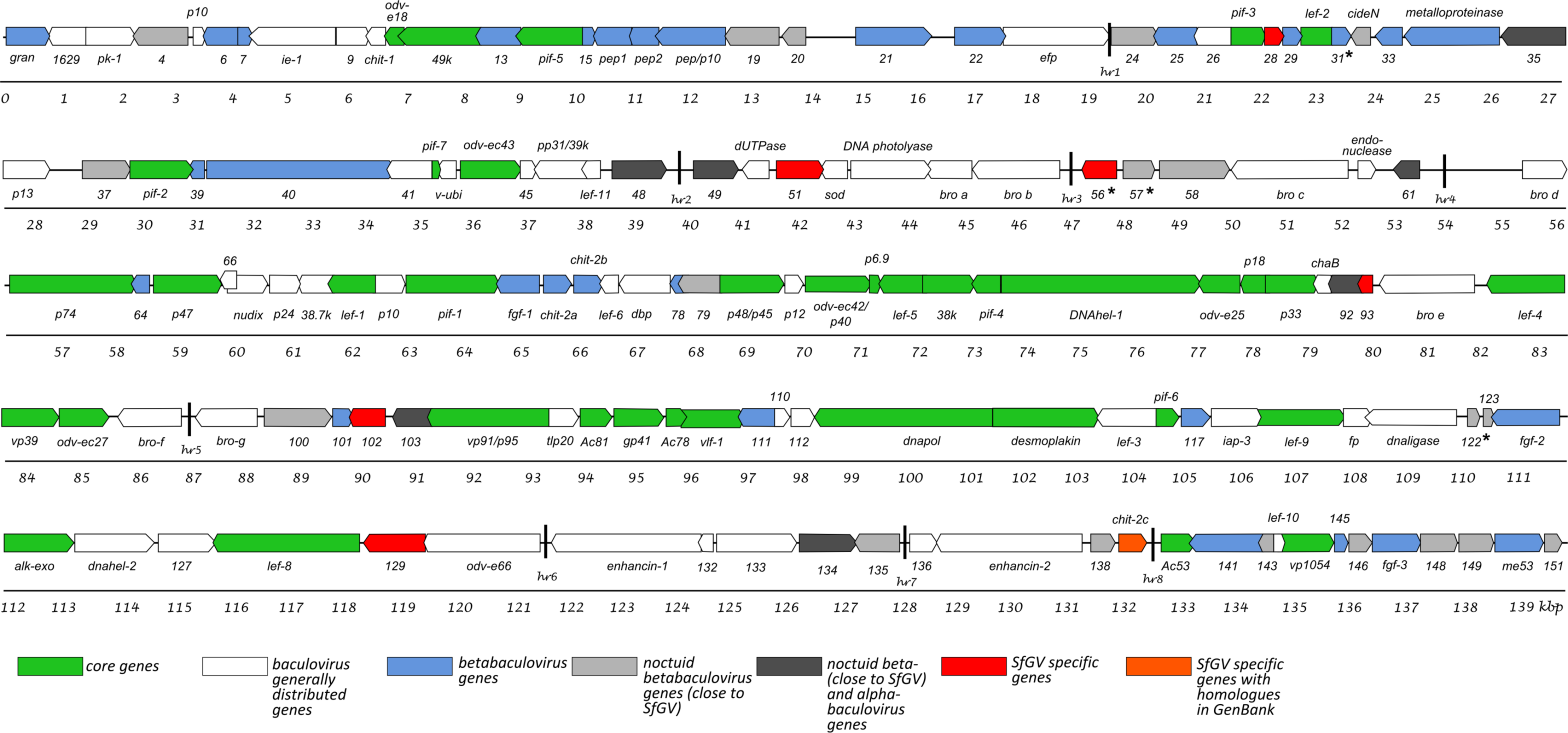
Linear map of SfGV-ARG. Arrows represent orientations of predicted ORFs. Each ORF is designated by its number in the genome annotation. Those numbers that belong to well conserved baculovirus orthologs have been replaced by the particular descriptive names. ORFs categories are indicated in the figure with different colors. Transcription products corresponding to some ORFs (indicated by asterisks) were checked and detected by RT-PCR (S1 Appendix). Homologous repeat regions (*hrs*) are represented by black lines.

SfGV ARG complete genome was obtained by Illumina and Sanger dideoxy sequencing (GeneBank accession no. MH170055). It consists of 139,812 bp with an AT content of 53.7%, similar to the AT content found in VG008. A total of 151 ORFs coding for polypeptides at least 50 amino acids (aa) long were annotated (Fig 1 and S1 Table). As expected, 38 core genes described for the *Baculoviridae* family [10, 11] were found in SfGV ARG.

### ORF content comparison of SfGV-ARG vs SfGV VG008

All ORFs detected in SfGV-ARG were found in VG008, even though some of them were not annotated in the VG008 GenBank entry. Of these, only 44.4% (67 ORFs) were 100% identical. Overall, SfGV-ARG shows an average of 98.3% amino acid identity with VG008 and some *bro* genes stand out as the most dissimilar (*bro-e* share 82.3% identity). Similarly, *bro* genes were found to be highly divergent in studies of the genome diversity of BmNPV [12, 13] and AgMNPV [14].

SfGV ARG genome is 1,101 bp smaller than the Colombian VG008 isolate (140,913 bp). This difference is due to the lack of a VG008 ORF084 (*lef-7*) homolog in SfGV ARG and a few indel blocks throughout the genome (S1 Fig). Nucleotide sequence repeats known as homologous regions (*hrs*) are a common feature in baculovirus genomes. *Hr*s function as enhancers of early gene transcription [15] and are thought to play a role as origins of replication [16], as transcription enhancers and in homologous recombination. SfGV ARG contains the same 8 *hrs* detected in SfGV VG008. All of them are identical except *hr*s 2, 3 and 7 which share 98–99% identity with their homologs in VG008.

**Fig 2:**
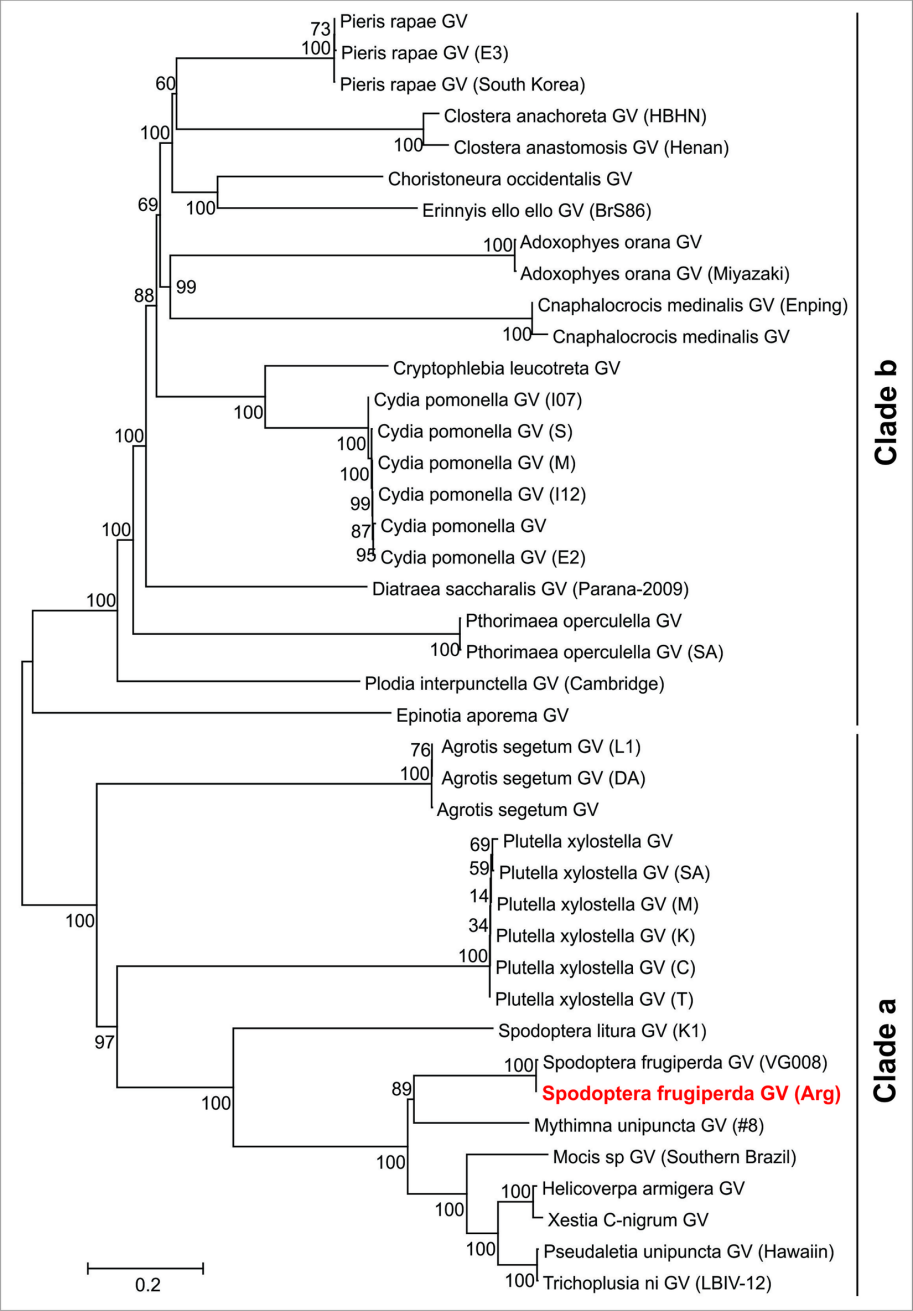
Betabaculovirus phylogenetic tree. Neighbor-Joining cladogram based on a concatemer of 37 core protein sequences obtained from 41 betabaculovirus genomes. The percentage of replicate trees in which the associated taxa clustered together in the bootstrap test is shown next to the branches. Virus genomes accession numbers are listed in S2 Table.

To date more than 40 complete betabaculovirus genomes have been sequenced. Phylogeny made with concatenated core genes (Fig 2) shows that the growing number of available genomes did not alter the previously detected separation in clades *a* and *b* of betabaculoviruses [17]. Focusing on the families of the order Lepidoptera from which betabaculoviruses were isolated, clade *a* contains noctuid-specific GVs with the exception of PlxyGV, which infects a plutellid family member. However, not all the noctuid-specific betabaculoviruses cluster together: *e*.*g*. within clade *a* AgseGV clade is separated from the SfGV clade. On the other hand, clade *b* is more diverse, it includes betabaculoviruses infecting insects of the families Tortricidae (ChocGV, AdorGV, CrleGV, CpGV and EpapGV), Pieridae (PrGV), Notodontidae (ClanGV, CaLGV), Sphingidae (ErelGV), Crambidae (CnmeGV, DisaGV), Gelichiidae (PhopGV) and Pyralidae (PlinGV).

More than a third of the 151 ORFs detected in SfGV ARG are only found in betabaculoviruses. These 56 ORFs include: 30 ORFs widely distributed in the genus, 19 ORFs found in noctuid betabaculoviruses closely related to SfGV, and 7 are SfGV specific (Fig 1 and S1 Table). Betabaculovirus specific genes are poorly studied. In this study we could detect selected mRNAs corresponding to ORF031 (group of betabaculovirus 30 ORFs), ORFs 057 and 122 (second group of 19 ORFs) and ORF056 (SfGV-specific), in dead *S*. *frugiperda* larvae infected with SfGV ARG (S1 Appendix). In an effort to better understand betabaculovirus diversification, Harrison and coworkers detected a group of genes that only occur in clade *a*—but not clade *b*—betabaculoviruses, based on the gene content analysis of Pseudaletia (Mythimna) sp. granulovirus #8 (MyunGV#8) genome [18]. Considering genes from SfGV that meet this criterion, this clade *a* list rises to 40 genes (S1 Table). In this study we sought to detect genes that were specific to the noctuid GVs closely related to SfGV (SpliGV, MyunGV, Mocis sp GV, HearGV, XcenGV, PsunGV and TniGV, S1 Table) and those present in this GV group plus any noctuid specific NPV. For the first group we found 19 ORFs: 004, 019, 020, 024, 032, 037, 057, 058, 080, 100, 122, 123, 135, 138, 143, 146, 148, 149 and 151 (colored in light grey in Fig 1 and S1 Table). For the second group we found seven ORFs: 035, 048, 049, 061, 092, 103 and 134 (coloured in dark grey in Fig 1 and S1 Table). ORF035 has homologs in some clade *a* betabaculoviruses and a match in a single member of alphabaculovirus genus, ORF165 of Leucania separata NPV, which in turn is most closely related to MyunGV#8 ORF41 [18]. ORF134 only matches with proteins from Crysodeixis includens NPV. ORFs 048, 049, 061, 092 and 103 could have been acquired by horizontal gene transfer as described for their homologs in SfGV VG008 (046, 047, 059, 089 and 099, respectively) [9].

### Genes with homologs in other virus families

**ORF020** is a 110 amino acid protein present in GVs closely related to SfGV according to blast searches, but homologs could also be found in *Heliothis virescens* and *Trichoplusia ni* ascoviruses using the Jackhmmer tool [19]. HvAV and SpliGV homologs include a longer N-terminal region absent in ORF020 and the rest of the baculovirus homologous proteins (S2 Fig).

**ORF026** codes for a 193 aa protein which has homologs in several GVs, two NPVs (Chfu-DEFMNPV and EppoNPV), several iridoviruses and Diadromus pulchellus ascovirus 4a. A conserved domain of unknown function was found in iridovirus homologs (DUF4433; pfam14487). This protein family is found in bacteria, archaea and eukaryotes, and are typically between 201 and 230 amino acids long. This family is distantly related to pfam01885, suggesting these may be ADP-ribosylases. Identity amongst all the homologous proteins is higher than 28%: *e*.*g*. ORF026 shares 31% identity with invertebrate iridovirus 6 homolog and 37.4% identity with Diadromus pulchellus ascovirus 4a protein (S3 Fig).

**ORF058** and **ORF0133** are 422 and 481 aa long homologs and share 26% identity and 40% similarity. Both ORFs match with proteins encoded in other GVs, one NPV (MacoNPV-B), entomopoxviruses and ascovirus. Two homologs were found in MyunGV, three in TniGV, Mocis sp. GV and HearGV, four in PsunGV, five in XcenGV, and one in AgseGV. Multiple alignment of these proteins show nine fully conserved cysteins and four prolines, as well (S4 Fig). Moreover, most of them contain an endonuclease superfamily domain (pfam 13930) at the C-terminal end. Thézé *et al*. reported the phylogeny of these proteins including homologs from insects and bacteria [20]. Recently, the homolog present in Mythimna separata entomopoxvirus was annotated as braconid endoparasitoid killer toxin (GenBank: BAO03247.1).

**ORF060** codes for a Ac79/Bm65 homolog (34% identity and 51% similarity with both gene products) and is a predicted member of the (GIY-YIG) nuclease superfamily. This protein is similar to bacterial UvrC and intron-encoded endonucleases which were found to participate in the nucleotide excision repair (NER) pathway for repair of UV-induced DNA damage and is responsible for the removal of bulky adducts such as cyclobutane pyrimidine dimers (CPDs) caused by UV. Tang et al. [21] showed that Bm65, present in BmNPV, may play a direct role in the repair of UV-induced DNA damage, or may directly affect the host’s ability to repair UV-induced damage. Homologs of these genes are present in some noctuid GVs and some NPVs and ascoviruses (S5 Fig).

**ORF100** codes for a putative 385 aa protein present only in some noctuid GVs genomes and also has a homolog in T. ni ascovirus 2c with which shares 20% identity (S6 Fig).

**ORF103** (VG008 ORF099) is well characterized by Cuartas *et al*. (2015). Homologs of this gene were not found in other betabaculoviruses but it has homologs in NPVs of the noctuid insects *S*. *frugiperda*, *Mamestra brassicae*, *Mamestra configurata*, *Helicoverpa armigera* and *Spodoptera litura*. Here we also found it has a homolog in Heliothis virescens ascovirus 3f, annotated as an iap-like protein [22].

**DNA ligase (ORF121)** is a special case because although it has homologs in eukaryotes, prokaryotes and several DNA viruses (including chordopoxvirus and phages), when it comes to DNA viruses that infect arthropods, only a few homologs were detected in GVs, a few NPVs -Lymantria dispar MNPV (Uniprot: A0A0D3QVZ8), Orgyia leucostigma NPV (Uniprot: B0FDP6) and Sucra jujuba NPV (A0A097P911), and invertebrate iridovirus 22 (Uniprot ID: S6DB12_9VIRU). Surprisingly, no hits in entomopoxvirus were found.

**ORF127** codes for a 333 aa putative protein with homologs in several baculovirus, ascovirus and poxvirus genomes. However no homologs were found in the entomopoxvirinae subfamily as in the case of DNA ligase.

### ORF066: Unannotated alpha and beta-baculovirus gene?

ORF066 gene partially overlaps the sequence coding the N-terminal region of *nudix* ORF, in opposite direction, and codes for a 74 aa protein. BlastP search yielded no matches; but tblastn analysis detected homologs of this gene in almost all of the GVs complete genomes in the region complementary of the nudix N-ter coding sequence. We found early CAKT initiator elements and a late DTAAG initiation motif upstream of this gene. In homologs of betabaculoviruses closely related to SfGV, TATA-box and CAKT elements were found as well. Due to the presence of *nudix* in several alphabaculoviruses we searched for putative homologs in the complementary sequence of the N-terminal region of this gene. A conserved peptide was also found coded in these genomes (S2 Appendix). Moreover, tblastn against transcriptomic databases detected putative NPV homologs from transcripts originated in the complementary chain of the *nudix* locus (not shown).

### Baculoviral F-box proteins

**Fig 3:**
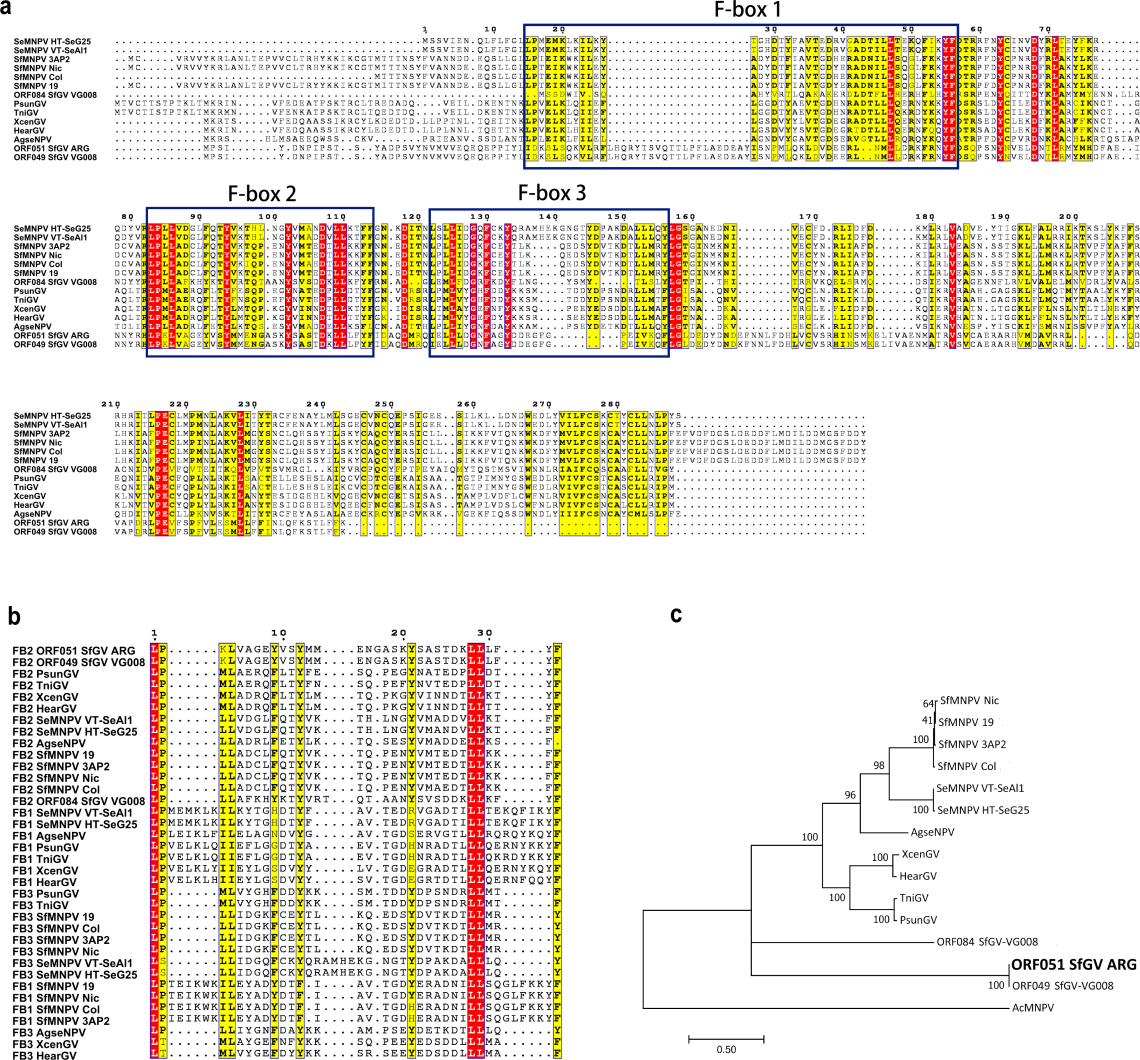
F-box proteins. (a) Multiple alignment of Lef-7 proteins from noctuid betabaculovirus and alphabaculovirus containing 3 F-box motifs, along with the paralogs found in SfGV genomes. F-box motifs are boxed. (b) Multiple alignment of F-box 1, 2 and 3 from proteins aligned in (a). (c) Phylogenetic analysis of baculovirus *lef-7* ORFs shown in (a) along with their SfGV ARG paralog ORF051 (and its homolog in VG008, ORF049), by Maximum Likelihood method. AcMNPV Lef-7 was used as an outgroup. Boostrap values (>50%, 100 replicates) are indicated for each node. Uniprot IDs of the proteins used are listed in S3 Table.

F-box proteins are components of the SCF (Skp1–Cullin–F-box) complex, a major type of E3 ubiquitin ligase multiprotein complexes, which mediate ubiquitination of proteins targeted for degradation by the 26S proteasome. In the SCF complex the F-box protein interacts through the F-box motif with the Skp1 protein and mediates substrate specificity through its second protein–protein interaction motif, leading to substrate polyubiquitination [23, 24]. Baculoviruses encode F-box proteins, including Lef-7 and Ac12 homologs. AcMNPV Lef-7 is a replication factor involved in the manipulation of the DNA Damage Response (DDR) of the host to optimize viral replication. AcMNPV Lef-7 was shown to be an F-Box protein which interacts with host S-phase kinase-associated protein (Skp1) suggesting that it plays a role in substrate recognition component of Skp1/Cullin/F-Box (SCF) complexes for targeted protein polyubiquitination [25]. Recently, ac12, which has few homologs in group I NPVs, was also demonstrated to interact with Skp1 [26]. Lef-7 homologs are present in several group I and II alphabaculovirus and in a few GVs specific for noctuid hosts: PsunGV, TniGV, XcenGV, HearGV, and SfGV VG008 (ORF084). When comparing the SfGV genomes VG008 vs ARG, VG008 ORF084 was the only ORF not found in SfGV ARG. We hypothesized that ORF051 could be a *lef-7* homolog due to a remote match with baculovirus Lef-7 in a blastP search. ORF051 codes for a 287 aa protein, homologous to ORF049 in VG008. Detailed analysis of these proteins along with other Lef-7 proteins found in GVs and some *Spodoptera* spp specific NPVs detected 3 putative F-box domains, in contrast to the unique F-box domain detected in AcMNPV Lef-7. The three domains contain a conserved Leu-Pro pair that was demonstrated to be essential in AcMNPV Lef-7 F-box for binding to *Spodoptera frugiperda* Skp1. Alignment of ORF051 with other Lef-7 sequences denotes that SfGV ORF051/ORF049 and VG008 Lef-7 seem to have lost two of the three domains, keeping the highly conserved central F-box domain (Fig 3A). Alignment of the three repeated motifs pointed at conserved Leu-Pro residues followed by highly conserved hydrophobic (mainly Leu) and aromatic (Tyr and Phe) residues (Fig 3B). This motif coincides with the recently reported for the two homologs of *lef-7* present in Mythimna unipuncta NPV [27]. There are six protein families in the F-box clan (CL0271) in the Pfam database [28] including F-box, F-box-like, PRANC and Elonguin A families. These F-box families are variable in size, ranging from 48 to 116 amino acids and inspection of their Pfam HHM logos permitted to visualize a variable domain with a general pattern consisting of Leu-Pro followed by Leu/Ile and Phe/Tyr positions. F-box proteins usually contain a carboxy-terminal protein-protein interaction domain beyond the F-box domain. The proteins analyzed in Fig 3 do not resemble AcMNPV Lef-7 C-ter, as it emerges from BlastP and HMMER searches. However, alignment examination showed that this group of proteins contains a leucine and cysteine rich region, resembling F-box proteins in which the F-box domain is followed by leucine reach repeats (LRR). Proteins with more than one F-box domain are reported in Pfam [28]. The functionality of three putative F-box domains in the viruses analyzed here should be demonstrated experimentally.

Phylogeny inferred from sequences analyzed in Fig 3C shows that Lef-7 proteins from *Spodoptera frugiperda* and *S*. *exigua* NPVs along with XcenGV, HearGV, TniGV and PsunGV form a monophyletic clade, distantly related to SfGV proteins. Despite the low resolution of the nodes connecting SfGV proteins, branch lengths show a high divergence between ORF084 and its paralog ORF049/051.

### Photolyase

**Fig 4:**
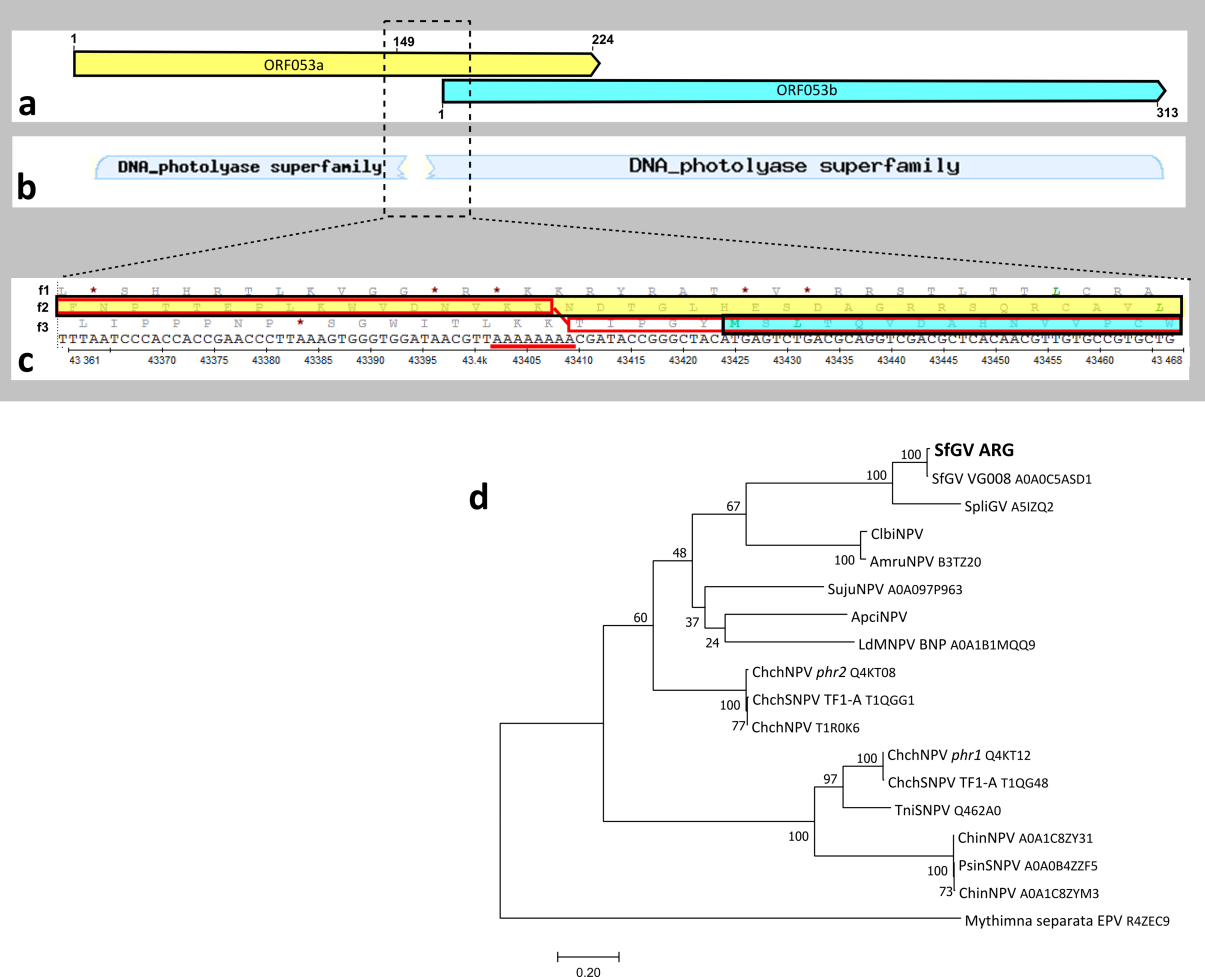
SfGV ARG Photolyase. (**a**) Schematic representation of two overlapping ORFs predicted automatically. (**b**) Homology to photolyase fragments obtained by BlastX. (**c**) Nucleotide sequence of the direct DNA strand of photolyase locus fragment and three reading frames f1, f2 and f3. Automatically detected ORFs 053a and 053b are shaded in yellow and blue in accordance with the schematic in panel (**a**). The translation product of combined f2 and f3 is boxed in red and the 8 consecutive adenine residues where frameshift would occur are underlined. (**d**) Phylogenetic analysis of baculovirus photolyases by Maximum Likelihood method. For SfGV ARG, ClbiNPV and ApciNPV photolyase we considered the complete polypeptides obtained after translation of combined frames (S3 Appendix). Uniprot IDs of the rest of the proteins are indicated. Boostrap values (>50%, 100 replicates) are indicated for each node. The sequence of Mythimna separata entmopoxvirus (MySEV) photolyase was included as an outgroup.

Photolyases are enzymes capable of repairing UV damaged DNA. Photolyase genes were found in some NPVs and in two granuloviruses: SpliGV and SfGV. Photolyase DNA locus encompasses 1402 nucleotides in SfGV ARG. It is identical to SfGV VG008 photolyase locus except for 9 substitutions and 1 nucleotide insertion. The insertion is located in a poly-A tract: 7 consecutive adenine residues in SfGV VG008 expanded to 8 A's in SfGV ARG. According to automatic ORF detection after this frameshift mutation the photolyase is split into two non-overlapping ORFs in SfGV ARG, which were annotated as ORFs 053a and 053b, respectively (Fig 4A). In contrast, SfGV VG008 encodes a single 1401 bp ORF coding for a 466 amino acid protein with homology to photolyases. Several Sanger dideoxy sequencing reactions were performed in order to discard errors and confirm that there is indeed a stretch of 8 consecutive A residues in SfGV ARG as a consequence of the insertion of one A. Typical blastP searches with ORFs 053a and 053b produced hits with photolyases. ORF 053a codes for a 224 amino acid product and has partial homology to photolyases: amino acids 1 to 149 match with first 149 amino acids of SfGV VG008 photolyase, whereas no hits were found with residues 150 to 224. On the other hand, ORF053b coding for 313 amino acid polypeptide matches completely with the last 313 amino acids of SfGV VG008 photolyase. Inspecting the whole locus we realized that when one A from the poly-A tract is eliminated, the two ORFs merge into a single ORF coding for a 466 aa protein which is 98,9% identical to its Colombian homolog. To further investigate this region, we performed a blastX analysis which uses a DNA sequence as a query and after translating the query in the six possible frames (three frames for each DNA strand) it executes a blast search against the protein database. BlastX does not look for the presence of ORFs beginning with a methionine codon; it just uses translation products between stop codons. This indicated that the product of conceptual translation which matches completely with the SfGV VG008 photolyase starts in frame with the predicted ORF053a (reading frame 2, f2) and then switches to the frame encoding ORF053b (reading frame 3, f3) and including 5 amino acids (TIPGY) encoded before the beginning of this ORF (Fig 4B and 4C). Interestingly, this conceptual frameshift lies within the above mentioned poly-A tract, where two consecutive Lys residues are coded. Poly-A tracts resemble ribosome “slippery” sequences that have been associated with programmed translational frameshift (PRF) [29]. It is difficult to predict which version of the photolyase is actually expressed: either the products of the automatically detected ORFs (with partial homology to photolyase for ORF053a) or the complete photolyase which is encoded in combination of two frames, that would imply a frameshift mechanism. Experiments that should be conducted to answer this question are not in the scope of this whole genome analysis study.

In order to explore if this feature was unique to SfGV ARG we examined photolyase genes coded by other baculoviruses. Notably, SfGV ARG is not the only baculovirus encoding an apparent split photolyase. Clanis bilineata NPV was reported to encode two consecutive ORFs originated by a 1bp deletion respect to the Chrysodeixis chalcites NPV *phr* (photolyase) homolog [30]. However, blastX search of the region encompassing both ORFs reveals that the photolyase continues in the *phr1* frame beyond this annotated ORF, in the ‘intergenic’ region between *phr1* and *phr2* (Fig A in S3 Appendix). A similar situation occurs in Apocheima cinerarium NPV, where two partially overlapping ORFs encode a complete photolyase upon frameshift within a poly-T tract, similar to the SfGV ARG case (Fig B in S3 Appendix). On the other hand, gene fusions and gene fissions are frequently found in studies of different isolates of the same virus: *e*.*g*., *pe-38*, *he65*, *ag144*, and *bro-c* were observed as single ORFs or divided into two independent ORFs in different isolates of AgMNPV [13]; AcMNPV WP10 was found to encode genes that appear as two different ORFs in C6 clone [31].

There are few reports on translational frameshifting events in baculovirus infected cells. Morikawa *et al*. showed that frameshifting occurred efficiently in insect cells when infected with a recombinant baculovirus carrying the *gag* and part of the *pol* gene of feline immunodeficiency virus [32]. In addition, Braunagel *et al*. proposed a frameshift mechanism to explain the presence of ODV-E35 in AcMNPV infected cells [33]. Ocurrence of frameshifting during translation of SfGV ARG photolyase requires experimental confirmation.

Phylogeny of baculovirus photolyase proteins was performed using the SfGV ARG 466 aa hypothetical protein encoded by the combination of two frames (Fig 4D). The same correction was considered for ApciNPV and ClbiNPV homologs (S3 Appendix). Two major groups were observed: one of them containing ChchNPV PHR1 related proteins and the other one containing ChchNPV PHR2, which includes a clade with the 3 betabaculovirus photolyases (SfGV ARG, SfGV VG008 and SpliGV). It is worth mentioning that in complementation assays ChchNPV PHR2 was able to complement a DNA repair deficient *Escherichia coli* strain showing that ChchNPV PHR2 is an active CPD photolyase whereas ChchNPV PHR1 could not rescue this deficiency [34].

## Conclusions

**Fig 5:**
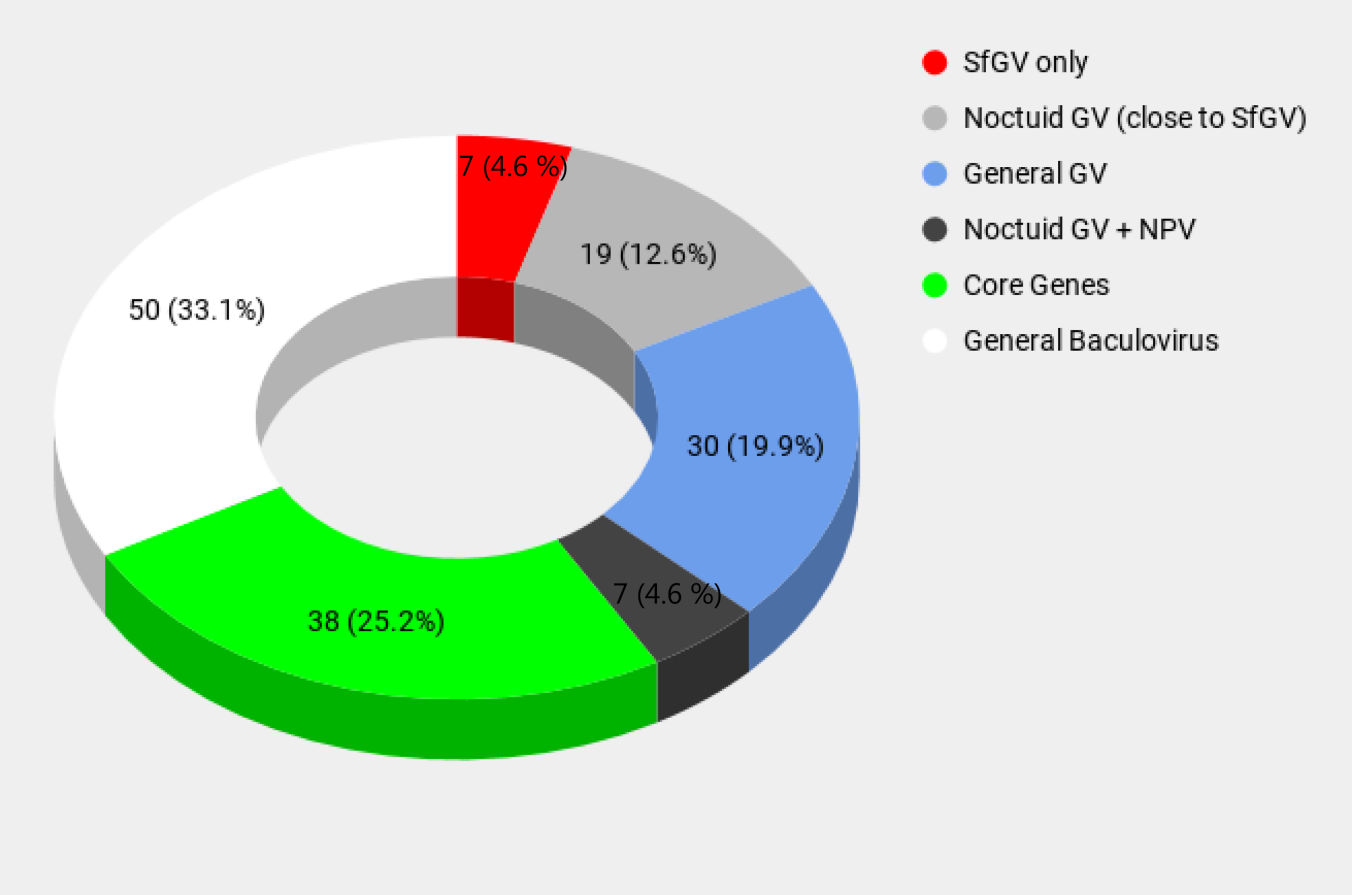
Percentage of ORFs in SfGV ARG according to their classification. Slices of the pie chart are colored according to ORFs classification depicted in Fig 1.

This work presents the genomic sequence of a new isolate of SfGV, SfGV ARG, native to Argentina. Of 151 ORFs, the presence of 38 core genes is a signature of the *Baculoviridae* family. Regarding the *Betabaculovirus* genus there are 56 genes with no homologs in NPVs. Of these, 30 genes are widely distributed in the genera, but there is an interesting proportion of genes only present in betabaculoviruses closely related to SfGV (19 ORFs, 12.6%). Moreover, summing up these genes with 7 genes also present in some noctuid specific NPVs, it can be noted that 26 genes (17.2%) might be related to a specific adaptation for noctuid hosts (Fig 5). Also, analysis of its gene content revealed that SfGV ARG lacks the *lef-7* homolog present in the VG008 isolate (ORF084). However, a remote paralog of this gene was found (ORF051), present in VG008 as well (ORF049). Detailed analysis of these genes detected 3 F-box motifs in a group of viruses including GVs and *Spodoptera spp*. NPVs, all infecting noctuid hosts. F-box proteins are widely distributed and highly diverse. Viral F-box proteins are difficult to detect by bioinformatics: conserved motif detection engines do not detect them, probably because sequences used for constructing HMM profiles include mainly F-box proteins from eukaryotes and only a few from viruses. Moreover, the F-box consensus has no strictly invariant residues, and contains gaps, making it difficult to detect them due to little conservation. However, there is experimental evidence for the interaction of some viral F-box proteins with Skp1, as is the case of AcMNPV Lef-7 [25] or the silencing suppressor protein P0 of two Arabidopsis-infecting poleroviruses which interacts by means of a conserved minimal F-box motif with *Arabidopsis thaliana* ortholog of S-phase kinase related protein 1 (Skp1) [35]. In both cases, the LP amino acid pair was found to be essential for the interaction with Skp1. Split ORFs are a common feature found in homologous genes in different isolates of a virus. SfGV ARG codes for two ORFs with partial homology to photolyase, feature also found in two other baculoviruses. But also we found a complete “hidden” photolyase coded in a combination of two frames. It is not known whether this protein is expressed as one protein, two smaller proteins or if it is not expressed at all. Photolyases are genes that belong to the accessory genome in baculoviruses, which allows more plasticity owing to the fact that they are non-essential genes [20]. Therefore, the photolyase activity, although not essential, could serve to improve its fitness. In this way, it is worth to mention that this accessory activity might be complemented by ORF060 which is involved in UV-damaged DNA repair as was demonstrated for its homolog in BmNPV. Although the expression of the complete photolyase could occur after a +1 PRF, this process remains to be experimentally demonstrated.

## Materials and methods

### Insects, virus and viral DNA purification

The Argentinean isolate of SfGV, SfGV ARG was obtained from dead *S*. *frugiperda* larvae and stored in IMyZA, INTA, Castelar, Argentina. For virus amplification, a laboratory *S*. *frugiperda* colony was established in IBBM, La Plata. SfGV ARG was amplified in 3 day old larvae fed with virus contaminated artificial diet and maintained individually at 26°C, 12:12 h (light:dark) photoperiod until death or pupation. Dead larvae were homogenized in ddH_2_O, gauze filtered and resuspended in 0,1% SDS. Cell debris were eliminated by low speed centrifugation (1000g, 2 min) and supernatant with viral OBs was centrifuged at 11000 g. OBs were washed twice with ddH_2_O and resuspended in ddH_2_O. OBs were loaded on to a discontinuous 35–60% (w/w) sucrose gradient and centrifuged at 20000 rpm 1 h and 4°C in SW41 rotor (Beckman). The band containing the OBs was removed and washed twice in ddH_2_O. Finally the viral pellet was resuspended in ddH_2_O. Purified OBs were dissolved by alkaline lysis and DNA extraction was performed as described previously [36].

### Genomic DNA sequencing and analysis

SfGV ARG genomic DNA was sequenced on Illumina HiSeq2000 platform and assembled *de novo* at Macrogen Corporation (South Korea). Additional PCR and Sanger dideoxy sequencing were performed in order to complete unresolved or ambiguous genome regions. The complete genome sequence and annotation information was submitted to GenBank (accession number: MH170055). ORF finding and genome annotation was performed using UGENE [37]. Homology searches were done using blastP and HMMER. Percentage identities between homologous genes were obtained by global alignments with ClustalO [38] using default parameters. Early (E) and late (L) promoter motifs were screened within 150 bp upstream of the putative ORFs. E indicates the presence of a TATA-box (TATAW, TATAWAW, TATAWTW) and a CAKT mRNA start site; whereas L denotes a DTAAG sequence. Betabaculovirus phylogeny was inferred using 37 core proteins [9] from 40 betabaculovirus genomes (GenBank; October 2017), plus core proteins of SfGV ARG. Each core protein set was independently aligned using ClustalX program [39], with the following parameters: Pairwise alignment (Gap Open Penalty = 10, Gap Extension Penalty = 0.1, protein weight matrix: Gonnet 250); Multiple alignment (Gap Open Penalty = 10, Gap Extension Penalty = 0.05, protein weight matrix: Gonnet series). Then a concatamer was generated by addition of the complete individual alignments and phylogeny was inferred using MEGA 6 program [40] with the following parameters: Method = Neighbor-Joining; Bootstrap with 1000 replicates; Model = Amino (JTT); patterns among sites = Same (Homogeneous); rates among sites = Different (Gamma Distributed); gamma parameter = 0.7483; gap/ Missing data = pairwise deletion. Phylogenies were inferred using the Maximum Likelihood method based on the LG + G + I model for Lef-7 proteins and LG + G for photolyase proteins and conducted in MEGA7 [41].

## Supporting information

S1 Table ORF and hr location in SfGV-ARG genome and comparison with their homologues of closely related betabaculovirus. (PDF)

S2 Table Accession number list of genomes used in Fig 2. (PDF)

S3 Table Uniprot IDs of lef-7 homologs used in Fig 3. (PDF)

S1 Appendix RT-PCR detection of different SfGV ARG mRNAs in infected larvae. (PDF)

S2 Appendix ORF066 homologs in alpha- and betabaculovirus. (PDF)

S3 Appendix ClbiNPV and ApciNPV photolyase genes. (PDF)

S1 Fig Relative similarity plot. (PDF)

S2 Fig Multiple alignment of ORF020 homologs. (PDF)

S3 Fig Multiple alignment of ORF026 homologs. (PDF)

S4 Fig Multiple alignment of ORF058, ORF133 and their homologs. (PDF)

S5 Fig Multiple alignment of ORF060 homologs. (PDF)

S6 Fig Multiple alignment of ORF100 homologs. (PDF)
