# Supplementary material for: Genomic analysis of an Argentinean isolate of Spodoptera frugiperda granulovirus reveals that various baculoviruses code for Lef-7 proteins with three F-box domains
Source: PLoS One. 2018 Aug 22;13(8):e0202598. doi: 10.1371/journal.pone.0202598 (PMC6105029; doi:10.1371/journal.pone.0202598)
Supplement: S1 Table — (PDF) [file pone.0202598.s001.pdf]

S1 Table **ORF and hr location in SfGV-ARG genome and comparison with their homologues of closely related betabaculovirus.**

ORFs were numbered starting from granulin (ORF001) in a clockwise direction considering the ORForder according to the order of each ATG position in the genome.

Coloring resembles grade of conservation in family Baculoviridae (for color reference see Figure 1 in the main text).

Two overlapping ORFs (053 a and 053b) are indicated for Photolyase gene although protein length and aminoacid identity was anotatded considering the complete ORF after correcting the nucleotide sequence (see main text for more information).

	Position								Orthologues in other betabaculoviruses #ORF (%identity)										
ORF	Start		End	Gene name	Size (aa)	TATA box	Early INR	Late INR	SfGV VG008	SpliGV	Mocis sp GV	PsunGV-H	MyunGV#8	TniGV	HearGV	XecnGV	CpGV	Clade a (no clade b)	Notes
001	1	>	747	*granulin*	248	X		X	1 (100)	1 (90.3)	1 (96.4)	1 (96.4)	1 (94.8)	AOW41340 (96.4)	1 (96.4)	1 (96.4)	1 (85.1)		
002	1408	<	797	*pk1*	287	X	X	X	2 (100)	3 (64.5)	3 (70.4)	3 (68.4)	3 (80.1)	AOW41342 (68.4)	3 (69.3)	3 (68.6)	3 (43.6)		
003	1389	>	2252	*orf1629*	203		X	X	3 (100)	2 (44.2)	2 (48.4)	2 (49.7)	2 (59.5)	AOW41341 (49.2)	2 (52.5)	2 (51.7)	2 (29.7)		
004	3189	<	2293		298	X	X	X	4 (100)	-	4 (30.7)	4 (35.3)	4 (24.7)	AOW41343 (35.2)	4 (33.6)	4 (33.6)	-	x	
005	3286	>	3507	*p10*	73	X	X	X	5 (100)	4 (62.5)	5 (71.2)	5 (74.0)	5 (71.2)	AOW41344 (74.0)	5 (69.9)	5 (71.2)	5 (17.7)		
006	4073	>	4327		84				6 (100)	6 (27.3)	7 (46.4)	7 (54.8)	8 (66.8)	AOW41346 (52.4)	7 (54.8)	8 (56.0)	6 (21.2)		
007	4083	<	3514		189		X		7 (100)	5 (43.1)	6 (55.6)	6 (60.4)	7 (46.5)	AOW41345 (60.4)	6 (62.0)	7 (62.0)	4 (31.9)		
008	5759	<	4320	*ie-1*	478		X		8 (99.6)	7 (41.2)	8 (53.2)	8 (56.0)	9 (49.8)	AOW41347 (56.0)	8 (55.7)	9 (55.9)	7 (28.8)		
009	5775	>	6347		190		X	X	9 (99.5)	8 (38.7)	9 (56.4)	9 (60.3)	10 (62.0)	AOW41348 (60.3)	9 (61.1)	10 (60.0)	8 (30.6)		DUF884
010	6664	<	6365	*chit-1*	99				10 (100)	9 (65.7)	10 (79.8)	10 (83.8)	11 (76.8)	AOW41349 (83.8)	10 (82.8)	11 (83.8)	9 (46.5)		
011	6674	<	6931	*odv-e18*	85		X	X	11 (98.8)	10 (73.8)	11 (82.9)	11 (79.5)	12 (81.0)	n/a (79.5)	11 (77.1)	12 (77.1)	14 (57.8)		
012	6932	<	8296	*49k*	454			X	12 (100)	11 (48.7)	12 (67.7)	12 (71.2)	13 (79.2)	AOW41350 (71.0)	12 (69.3)	13 (68.4)	15 (42.0)		
013	8313	<	8990		225				13 (99.6)	12 (28.3)	13 (60.0)	13 (61.3)	14 (57.4)	AOW41351 (61.3)	13 (60.9)	14 (61.8)	-	x	RING finger (PF13923)
014	9010	<	10071	*pif5*	353		X	X	14 (99.2)	13 (55.1)	14 (70.0)	14 (68.3)	15 (67.6)	AOW41352 (68.6)	14 (67.4)	15 (69.4)	18 (56.6)		
015	10112	>	10324		70	X	X		15 (100)	-	15 (49.3)	15 (63.2)	16 (59.4)	AOW41353 (63.2)	15 (51.4)	16 (54.3)	19 (34.3)		DUF919
016	10336	<	10926	*pep-1*	196		X	X	16 (100)	15 (57.6)	16 (67.8)	16 (70.7)	18 (65.5)	AOW41354 (70.7)	16 (72.4)	17 (72.4)	20 (47.6)		
017	10962	<	11420	*pep-2*	152		X	X	17 (96.0)	18 (53.5)	17 (80.3)	17 (81.6)	19 (84.2)	AOW41355 (80.9)	17 (80.3)	18 (80.9)	23 (49.3)		
018	11441	<	12592	*pep/p10*	383		X	X	18 (99.2)	19 (57.9)	18 (75.9)	18 (77.3)	20 (78.3)	AOW41356 (77.3)	18 (76.4)	19 (76.2)	22 (54.7)		
019	12667	<	13524		285	X			19 (98.6)	-	22 (22.6)	22 (38.6)	23 (33.7)	AOW41360 (38.9)	22 (38.9)	23 (38.9)	-	x	
020	13661	<	13993		110	X	X		20 (100)	-	-	23 (49.1)	24 (54.6)	AOW41361 (49.1)	23 (56.4)	24 (56.4)	-	x	Tni ascovirus Q06VL3
021	14883	>	16181		432		X		21 (99.5)	22 (27.1)	23 (48.7)	24 (48.3)	26 (42.1)	AOW41362 (48.3)	24 (47.9)	25 (47.8)	-	x	
022	16600	>	17472		290	X	X		22 (100)	-	24 (29.9)	25 (31.1)	28 (36.7)	AOW41363 (31.9)	25 (28.3)	26 (29.4)	-	x	
023	17450	>	19297	*efp*	615		X		23 (99.2)	24 (32.0)	25 (53.8)	26 (54.8)	29 (54.4)	AOW41364 (54.9)	26 (54.6)	27 (54.4)	31 (32.5)		
hr1	19302	>	19350																
024	19325	>	20143		272	X			24 (99.3)	25 (22.0)	27 (23.5)	-	30 (25.3)	-	27 (26.7)	28 (25.7)	-	x	coiled coil
025	20140	<	20826		228		X		25 (99.1)	26 (45.1)	28 (55.8)	28 (56.1)	31 (59.9)	AOW41365 (56.1)	28 (56.6)	29 (56.3)	33 (32.6)		
026	20843	<	21424		193		X	X	26 (100)	-	-	29 (76.0)	32 (82.3)	AOW41366 (76.0)	29 (70.3)	30 (65.6)	34 (52.1)		iridovirus and ascovirus homologues
027	21442	>	22020	*pif-3*	192		X	X	27 (99.5)	27 (43.9)	29 (63.4)	30 (62.8)	33 (66.2)	AOW41367 (62.8)	30 (62.0)	32 (61.9)	35 (46.0)		
028	22027	>	22329		100		X	X	28 (100)	-	-	-	-	-	-	-	-		
029	22333	>	22668		111	X	X	X	29 (100)	31 (50.0)	31 (77.5)	32 (78.4)	35 (82.7)	AOW41368 (78.4)	32 (78.4)	34 (78.4)	39 (46.9)		coiled coil, bZIP
030	22671	>	23267	*lef-2*	198		X	X	30 (100)	32 (45.6)	32 (59.9)	33 (58.6)	36 (59.0)	AOW41369 (58.6)	33 (58.6)	35 (58.5)	41 (39.4)		
031	23200	>	23523		107		X		31 (100)	33 (44.4)	33 (26.0)	34 (30.3)	37 (44.7)	AOW41370 (30.4)	34 (32.6)	36 (32.6)	42 (25.9)		
032	23596	<	23871	*CIDE_N*	91	X	X	X	32 (100)	35 (33.8)	-	36 (60.7)	38 (56.0)	-	36 (56.0)	38 (56.0)	-	x	
033	24031	<	24441		136	X	X		33 (99.3)	36 (24.4)	34 (48.5)	37 (50.7)	39 (57.4)	AOW41372 (50.7)	37 (50.4)	39 (50.4)	45 (18.7)		CCDC14, 3 x coiled coil
034	24519	<	26138	*mpnase*	539	X	X	X	34 (99.6)	37 (39.2)	35 (48.8)	38 (52.8)	40 (49.0)	AOW41373 (52.8)	38 (52.8)	40 (56.7)	46 (31.9)		
035	26221	<	27312		363	X		X	35 (99.7)	-	36 (25.1)	40 (26.6)	41 (25.9)	-	39 (26.0)	42 (24.9)	-	x	LeseNPV ORF165
036	27393	>	28235	*p13*	280	X	X	X	36 (100)	38 (55.0)	37 (70.2)	41 (71.2)	42 (72.3)	AOW41374 (71.2)	40 (71.8)	43 (71.5)	47 (51.7)		
037	28848	>	29705	*hyp prot zn finger*	286			X	n/a (99.6)	-	-	42 (27.6)	-	AOW41375 (27.6)	41 (25.3)	44 (22.8)	-	x	Ring Finger + coiled coil
038	29717	>	30859	*pif-2*	380			X	37 (100)	39 (57.7)	40 (75.3)	43 (76.6)	43 (74.1)	AOW41376 (76.6)	42 (76.1)	45 (75.8)	48 (51.9)		
039	30852	<	31091		79	X		X	n/a (97.5)	40 (29.5)	41 (33.8)	44 (39.5)	44 (30.8)	AOW41377 (39.5)	43 (38.0)	46 (37.2)	49 (24.4)		
040	31113	>	34544		1093			X	38 (99.2)	41 (20.9)	42 (40.4)	45 (40.7)	45 (42.4)	AOW41378 (40.7)	44 (38.3)	47/48 (59.8/30.6)	50/51 (20.5/20.2)		SMC-N
041	34537	<	35220		227	X		X	39 (100)	42 (79.5)	43 (78.0)	47 (79.7)	46 (84.4)	AOW41380 (79.7)	45 (80.0)	50 (80.9)	52 (52.6)		DUF816, ac106/107
042	35268	>	35429	*pif-7*	53	X			40 (100)	43 (45.1)	44 (65.4)	48 (75)	47 (67.3)	AOW41381 (73.6)	46 (69.8)	51 (69.8)	53 (41.7)		DUF1443, ac110, pif
043	35452	<	35700	*v-ubi*	82	X		X	42 (100)	45 (89.5)	45 (96.1)	50 (94.7)	48 (93.5)	AOW41383 (93.5)	47 (96.1)	52 (97.4)	54 (79.3)		PF00240.21, ac35

044	35802	>	36857	*odv-ec43*	351	X	X	X	41 (100)	46 (53.4)	46 (69)	51 (73.4)	50 (74.6)	AOW41384 (72.9)	48 (71.1)	53 (71.1)	55 (41.9)		DUF673, PF05054.10
045	36874	>	37179		101		X	X	43 (99)	47 (41.3)	47 (71.3)	52 (71.9)	51 (66.3)	AOW41385 (69.7)	49 (70.1)	54 (69.1)	56 (27.9)		*ac108*
046	37215	<	38090	*pp31/39k*	291	X	X	X	44 (100)	48 (61.8)	48 (72.7)	53 (72.1)	52 (67.7)	AOW41386 (69.2)	50 (71.1)	55 (71.1)	57(26.5)		*ac36*
047	38080	<	38355	*lef-11*	91	X	X	X	45 (97.8)	49 (47.7)	49 (73.3)	54 (76.7)	53 (76.9)	-	51 (77.5)	56 (76.7)	58 (51.7)		ac37
048	38565	>	39527		320	X		X	46 (98.7)	-	50 (21.1)	55 (22.9)	57 (21.8)	AOW41387 (22.9)	52 (23.1)	57 (22.9)	-	x	
hr2	39668	>	39996																
049	40061	>	40852		263	X	X	X	47 (98.8)	-	50 (28.3)	55 (30.7)	57 (21.9)	AOW41387 (30.7)	52 (30.0)	57 (29.3)	-	x	
050	41017	>	41442	*dUTPase*	141	X	X	X	48 (100)	51 (77.9)	-	-	-	-	-	-	-		
051	41564	>	42427		287	X	X		49 (99.6)	-	-	-	-	-	-	-	-		*lef-7* paralogue
052	42443	<	42871	*sod*	142	X	X		50 (99.3)	-	52 (69.72)	64 (68.3)	58 (69.0)	AOW41394 (68.3)	63 (70.4)	68 (71.8)	59 (60.6)		*ac31*
053a	42961	>	43635	*DNA photolyase*	466	X			51 (98.9)	50 (76.6)	-	-	-	-	-	-	-		
053b	43424	>	44362																
054	44397	<	45155	*bro a*	238	X	X		52 (98.8)	114 (38.8)	-	162 (29.5)	-	-	-	130 (34.7)	-		Bro-N (PF02498), GIY-YIG-SF (PF13455)
055	45240	<	46772	*bro b*	510		X		53 (96.6)	-	-	66 (56.9)	-	AOW41396 (56.7)	133 (43.6)	131 (44.0)	-		Bro-N
hr3	46806	>	47210																
056	47238	<	47807		189	X		X	54 (99.5)	-	-	-	-	-	-	-	-		
057	47930	>	48484		184	X	X	X	55 (98.4)	-	54 (22.1)	70 (23.4)	60 (24.2)	AOW41400 (23.1)	65 (20.0)	71 (21.4)	-	x	
058	48615	>	49883	*pkt*	422	X	X	X	56 (99.8)	-	56 (47.5)	72 (50.1)	61 (50.8)	AOW41402 (50.1)	67 (50.8)	73 (51.6)	-	x	+ entomopoxvirus + ascovirus
059	49965	<	52070	*bro c*	701	X	X		57 (97.7)	-	-	-	62 (25.5)	-	-	-	-		Bro-N, colied coil
060	52241	>	52522	*endonuclease*	93	X			58 (100)	-	-	74 (62.9)	66 (60.2)	AOW41404 (62.9)	69 (63.7)	75 (62.6)	-		PF01541.22, GIY-YIG-SF +ascovirus + iridovirus
061	52969	<	53382		137	X	X	X	59 (99.3)	-	-	-	-	-	-	-	-	x	
hr4	53658	>	53906																
062	55266	>	56069	*bro d*	267	X	X	X	60 (99.6)	-	59 (52.1)	76 (55.6)	67 (52.9)	AOW41406 (56.0)	71 (55.4)	76 (55.8)	-		
063	55984	>	58224	*p74*	746		X		61 (99.9)	56 (41.9)	60 (66.5)	77 (67.7)	68 (64.3)	AOW41407 (67.7)	72 (68.4)	77 (68.4)	60 (41.3)		
064	58178	<	58450		90	X	X		62 (98.9)	57 (41.0)	61 (56.5)	78 (58.0)	-	AOW41408 (58.0)	73 (61.4)	-	62 (38.5)		
065	58522	>	59706	*p47*	394	X	X		63 (99.7)	58 (64.4)	62 (74.8)	79 (75.8)	70 (74.8)	AOW41409 (75.8)	74 (75.8)	78 (75.8)	68 (58.5)		
066	59757	<	59981		74		X		n/a (100)	n/a (26.9)	n/a (71.2)	n/a (71.2)	n/a (48.6)	n/a (71.2)	n/a (68.2)	n/a (51.3)	n/a (73.8)		new, unannotated baculovirus gene
067	59833	<	60516	*nudix*	227	X	X	X	64 (100)	59 (78.2)	63 (92.4)	82 (92.9)	71 (86.2)	AOW14412 (92.9)	77 (92.0)	79 (92.4)	69 (65.6)		
068	60581	>	61111	*p24*	176	X		X	65 (99.4)	60 (53.7)	64 (65.7)	83 (67.0)	72 (63.9)	AOW41413 (67.0)	78 (66.9)	80 (66.9)	71 (45.1)		ac129
069	61127	<	61648	*38.7k*	173	X	X		66 (98.8)	61 (26.8)	65 (44.4)	84 (50.3)	73 (45.8)	AOW41414 (50.3)	79 (52.5)	81 (50.0)	73 (22.1)		ac13
070	61650	<	62366	*lef-1*	238		X		67 (99.6)	62 (55.13)	66 (69.6)	85 (74.7)	74 (70.5)	AOW41415 (74.7)	80 (72.1)	82 (71.7)	74 (49.6)		ac14
071	62431	>	62952	*p10*	173		X	X	68 (99.4)	63 (38.9)	67 (61.4)	86 (54.8)	75 (58.4)	AOW41416 (54.8)	81 (59.9)	83 (59.2)	-		
072	62976	>	64601	*pif-1*	541		X	X	69 (100)	64 (42.6)	68 (60.0)	87 (59.8)	76 (58.3)	AOW41417 (59.8)	82 (60.4)	84 (60.1)	75 (42.2)		ac119
073	64639	<	65325	*fgf-1*	228		X		70 (100.0)	66 (30.8)	69 (39.4)	88 (42.3)	77 (42.7)	AOW41418 (42.7)	83 (44.4)	85 (44.4)	76 (21.4)		
074	65396	>	65884	*chit-2a*	162	X	X	X	71 (98.8)	68 (36.3)	71 (18.4)	90 (28.0)	79 (45.9)	AOW41419 (28.0)	85 (28.0)	87 (27.4)	79 (28.0)		ac150
075	65952	>	66431	*chit-2b*	159		X	X	72 (98.7)	68 (37.0)	71 (39.0)	90 (41.2)	80 (40.1)	AOW41419 41.2()	85 (38.6)	87 (34.6)	79 (28.0)		ac150
076	63428	<	66727	*lef-6*	99				73 (100.0)	69 (40.0)	72 (63.5)	91 (59.4)	81 (67.0)	AOW41420 (59.4)	86 (60.8)	88 (61.9)	80 (37.5)		ac28
077	66782	<	67627	*dbp*	281	X	X		74 (100.0)	70 (24.3)	73 (37.6)	92 (38.6)	82 (41.1)	AOW41421 (38.6)	87 (39.3)	89 (40.0)	-		ac25
078	67681	<	67962		93				n/a (98.9)	71 (42.9)	74 (48.5)	93 (50.0)	83 (50.0)	AOW41421 (12.1)	88 (50.0)	89a (13.2)	-		
079	68503	>	69621	*p48/p45*	372		X	X	75 (100.00)	73 (65.0)	76 (80.1)	95 (80.4)	85 (82.3)	AOW41424 (80.4)	90 (82.5)	91 (83.3)	83 (57.7)		ac103
080	67833	<	68504		223		X	X	76 (99.1)	72 (20.4)	75 (43.8)	94 (42.5)	84 (50.0)	AOW41423 (42.5)	89 (39.5)	90 (40.0)	-	x	
081	69629	>	69964	*p12*	111		X	X	77 (100.0)	74 (54.7)	77 (60.9)	96 (65.5)	86 (66.4)	AOW41425 (65.5)	91 (63.6)	92 (63.6)	84 (38.6)		ac102
082	70016	>	71134	*odv-ec42/p40*	372		X	X	78 (99.5)	75 (63.5)	78 (77.1)	97 (78.0)	87 (80.6)	AOW41426 (78.0)	92 (78.7)	93 (77.3)	85 (51.4)		ac101
083	71157	>	71333	*p6.9*	58		X	X	79 (100.0)	-	79 (87.5)	98 (85.7)	88 (73.7)	-	93 (83.3)	94 (83.9)	86 (60.9)		ac100
084	72077	>	72991	*38k*	304		X	X	80 (100.0)	77 (56.6)	81 (64.0)	100 (65.8)	90 (71.3)	AOW41428 (65.8)	95 (67.6)	96 (67.9)	88 (47.2)		ac98
085	71372	<	72154	*lef-5*	260		X		81 (100.0)	76 (60.7)	80 (73.5)	99 (73.8)	89 (79.0)	AOW41427 (73.8)	94 (75.0)	95 (70.4)	87 (55.8)		ac99
086	73465	>	76956	*dnahel-1*	1163	X	X		82 (99.7)	80 (65.7)	83 (75.4)	102 (76.1)	92 (77.5)	AOW41430 (76.1)	97 (75.2)	98 (75.1)	90 (38.3)		ac95
087	72993	<	73466	*pif-4*	157				83 (100.0)	79 (53.9)	82 (76.4)	101 (77.7)	91 (78.3)	AOW41429 (77.7)	96 (77.7)	97 (77.1)	89 (47.4)		ac96
088	77017	<	77676	*odv-e25*	219	X	X	X	85 (100.0)	81 (67.3)	84 (79.9)	103 (81.2)	93 (78.4)	AOW41433 (81.2)	98 (82.7)	99 (81.3)	91 (64.8)		ac94
089	78130	>	79041	*p33*	303		X		86 (100.0)	83 (51.8)	86 (70.9)	105 (73.3)	95 (72.5)	AOW41435 (73.3)	100 (71.7)	101 (72.1)	93 (48.6)		ac92
090	77733	<	78209	*p18*	158		X	X	87 (100.0)	82 (52.9)	85 (62.0)	104 (66.5)	94 (76.0)	AOW41434 (66.5)	99 (67.1)	100 (62.3)	92 (43.0)		ac93
091	79027	<	79266	*chaB*	79	X	X	X	88 (98.7)	84 (49.3)	87 (75.3)	106 (68.4)	96 (78.5)	AOW41436 (68.4)	103 (73.4)	102 (69.6)	-		ac60

092	79290	<	79790		166				89 (99.4)	-	-	132 (33.3)	-	AOW41460 (33.3)	131 (35.0)	128 (36.4)	-	x	
093	79807	<	80013		68	X	X		n/a (98.5)	-	-	-	-	-	-	-	-		
094	80213	<	81796	*bro e*	527	X	X	X	90 (82.3)	111 (49.5)	-	137 (49.6)	21 (42.0)	AOW41465 (50.2)	54 (44.0)	60 (48.9)	-		bro-N, coiled coil
095	82088	<	83413	*lef-4*	441	X	X	X	91 (99.3)	86 (52.2)	90 (65.5)	114 (67.7)	100 (68.0)	AOW41442 (67.9)	112 (67.0)	110 (66.7)	95 (48.6)		ac90
096	83469	>	84443	*vp39*	324	X	X	X	92 (100.0)	87 (58.0)	91 (73.4)	115 (71.6)	101 (74.7)	AOW41443 (71.9)	113 (69.4)	111 (70.1)	96 (38.9)		ac89
097	84516	>	85388	*odv-ec27/odv-e27*	290		X		93 (100.0)	88 (66.2)	92 (79.9)	116 (80.9)	102 (80.9)	AOW41444 (80.9)	114 (80.2)	112 (80.2)	97 (44.0)		ac144
098	85605	<	86717	*bro f*	370	X	X	X	94 (100.0)	89 (37.1)	93 (51.7)	117 (54.0)	103 (59.5)	AOW41445 (54.0)	116 (52.6)	113 (52.9)	-		
hr5	86811	>	86902																
099	87060	<	88124	*bro g*	354	X	X	X	95 (100.0)	-	95 (66.7)	118 (67.4)	104 (67.1)	AOW41446 (67.4)	117 (69.8)	114 (70.1)	-		
100	88247	>	89401		385	X	X		96 (99.7)	-	96 (53.4)	119 (57.98)	105 (55.97)	AOW41447 (57.78)	118 (57.34)	115 (57.62)	-	x	ascovirus
101	89463	>	89837		124	X	X	X	97 (99.2)	90 (44.0)	98 (64.17)	120 (70.00)	106 (66.94)	AOW41448 (70.0)	119 (71.67)	116 (70.83)	100 (37.36)		
102	89819	<	90409		196	X	X		98 (98.98)	-	-	-	-	-	-	-	-		
103	90612	<	91208		198		X	X	99 (97.47)	-	-	-	-	-	-	-	-	x	NPV and ascovirus homologues
104	93359	>	93904	*tlp20*	181	X	X		100 (99.44)	93 (33.33)	100 (64.63)	123 (64.15)	109 (68.07)	AOW41451 (64.15)	122 (67.53)	119 (66.88)	102 (21.82)		ac82
105	91234	<	93390	*pif-8*	718		X	X	101 (99.86)	92 (43.76)	99 (60.77)	122 (59.71)	108 (62.46)	AOW41450 (59.71)	121 (61.33)	118 (60.59)	101 (36.16)		ac83, vp91/p95
106	93955	>	94530	*Ac81*	191	X	X	X	102 (100.00)	94 (63.10)	101 (77.54)	124 (78.07)	110 (82.63)	AOW41452 (78.07)	123 (78.61)	120 (78.61)	103 (55.26)		ac81
107	94559	>	95443	*gp41*	294	X	X		103 (100.00)	95 (69.37)	102 (73.10)	125 (73.79)	111 (78.77)	AOW41453 (73.79)	124 (75.17)	121 (75.17)	104 (49.83)		ac80
108	95517	>	95828	*Ac78*	103		X	X	104 (100.00)	96 (35.16)	103 (54.37)	126 (56.31)	112 (56.70)	AOW41454 (56.31)	125 (57.84)	122 (56.86)	105 (29.63)		ac78
109	95800	>	96906	*vlf-1*	368	X	X	X	105 (100.00)	97 (66.03)	104 (76.32)	127 (79.11)	113 (80.05)	AOW41455 (79.11)	126 (77.90)	123 (77.90)	106 (56.13)		ac77
110	97473	>	97730		85	X	X	X	106 (100.00)	99 (81.18)	106 (90.59)	129 (88.24)	115 (91.76)	AOW41457 (88.24)	128 (89.41)	125 (89.41)	107 (58.33)		DUF843; ac76
111	96896	<	97567		223		X		107 (99.55)	98 (39.52)	105 (60.47)	128 (60.82)	114 (61.27)	AOW41456 (60.82)	127 (61.27)	124 (60.12)	-	x	
112	97770	>	98207		145		X	X	108 (99.31)	100 (44.83)	107 (64.10)	130 (71.72)	116 (52.45)	AOW41458 (71.72)	129 (69.66)	126 (69.66)	108 (34.03)		DUF1160; ac75
113	98247	<	101447	*dnapol*	1066	X	X		109 (99.72)	101 (62.37)	109 (73.22)	138 (72.43)	117 (75.05)	AOW41466 (72.72)	134 (72.54)	132 (72.63)	111 (51.78)		ac65
114	101446	>	103359	*desmoplakin*	637		X		110 (99.84)	102 (34.80)	110 (51.06)	139 (54.05)	118 (53.00)	AOW41467 (54.05)	135 (53.72)	133 (53.72)	112 (24.85)		ac66
115	104404	>	104823	*pif-6*	139				111 (100)	104 (45.31)	112 (57.35)	141 (68.38)	120 (65.22)	-	137 (66.18)	135 (60.00)	114 (40.48)		ac68
116	103386	<	104438	*lef-3*	350	X	X		112 (100)	103 (39.31)	111 (48.69)	140 (46.90)	119 (49.42)	AOW41468 (46.31)	136 (46.09)	134 (45.80)	113 (26.14)		ac67
117	104860	>	105354		164			X	113 (100)	105 (28.86)	113 (37.20)	142 (40.49)	121 (37.27)	AOW41470 (40.49)	138 (38.04)	136 (39.26)	-		
118	105415	>	106284	*iap-3*	289	X	X		114 (99.31)	106 (36.55)	114 (56.20)	143 (55.52)	122 (61.34)	AOW41471 (55.87)	139 (55.96)	137 (55.76)	116 (33.59)		
119	106176	>	107807	*lef-9*	543			X	115 (99.82)	107 (68.40)	115 (79.80)	145 (81.22)	123 (83.57)	AOW41473 (81.22)	140 (80.82)	139 (81.02)	117 (63.82)		ac62
120	107791	>	108303	*fp*	170		X		116 (100)	109 (52.86)	116 (79.72)	146 (82.86)	124 (82.31)	AOW41474 (85.03)	141 (84.35)	140 (82.31)	118 (49.34)		ac61
121	108275	<	109879	*dnaligase*	534	X	X	X	117 (100)	110 (52.6)	117 (67.4)	148 (69.8)	125 (71.6)	AOW41476 (69.8)	142 (68.4)	141 (68.4)	120 (43.8)		poxvirus
122	110061	>	110261		66	X	X		118 (100)	-	118 (56.2)	149 (59.0)	126 (59.1)	AOW41477 (59.0)	143 (53.0)	142 (53.0)	-	x	
123	110322	>	110522		66	X	X	X	119 (100)	-	119 (60.0)	150 (67.7)	127 (63.6)	AOW41478 (67.7)	144 (67.7)	143 (67.7)	-	x	
124	110558	<	111721	*fgf-2*	387	X			120 (100)	116 (42.4)	120 (51.94)	151 (53.0)	128 (60.85)	AOW41479 (53.03)	145 (51.4)	144 (50.9)	123 (24.5)		ac32
125	111847	>	113064	*alk-exo*	405	X	X	X	121 (99.5)	117 (45.3)	121 (60.0)	152 (63.3)	129 (65.7)	AOW41480 (63.28)	146 (59.8)	145 (59.1)	125 (41.2)		ac33
126	113109	>	114527	*dnahel-2*	472	X	X		122 (99.6)	118 (55.1)	122 (65.7)	153 (65.4)	130 (66.3)	AOW41481 (65.6)	147 (66.30)	146 (65.4)	126 (48.2)		
127	114623	>	115624		333	X	X		123 (99.7)	120 (36.7)	123 (49.7)	154 (57.4)	131 (56.6)	AOW41482 (57.4)	148 (57.2)	147 (51.80)	-		DUF2661, ac112/113, ascovirus, chordopoxvirus
128	115644	<	118223	*lef-8*	859			X	124 (99.5)	121 (70.5)	124 (81.05)	155 (82.2)	132 (83.4)	AOW41483 (82.1)	149 (83.0)	148 (82.8)	131 (64.5)		ac50
129	118381	<	119397		303				125 (98.5)	-	-	-	-	-	-	-	-		
130	119444	<	121468	*odv-e66*	675		X	X	126 (99.55)	125 (20.7)	125 (65.7)	156 (71.9)	133 (72.8)	AOW41484 (71.7)	150 (71.3)	149 (71.3)	37 (41.7)		ac46
hr6	121521	>	121705																
131	121742	<	124345	*enhancin-1*	867	X	X	X	127 (99.3)	-	126 (29.2)	159 (31.7)	135 (45.4)	AOW41487 (31.6)	152 (45.7)	152 (45.7)	-		
132	124381	<	124581		66	X	X		n/a (92.4)	126 (50.0)	128 (53.3)	163 (50.0)	136 (65.1)	AOW41490 (50.0)	-	160 (46.9)	-		ac111
133	124627	>	126072		481		X	X	128 (96.5)	-	129 (54.1)	164 (54.5)	137 (56.0)	AOW41491 (54.5)	160 (57.6)	161 (58.0)	-	x	NUC superfamily, entomopox, ascovirus
134	126126	>	127130		334	X	X	X	129 (98.8)	-	-	-	-	-	-	-	-	x	
135	127157	<	127918		220		X		130 (100.0)	-	130 (34.8)	165 (42.25)	138 (40.0)	AOW41492 (42.25)	161 (40.9)	162 (40.9)	-	x	
hr7	127813	>	128128																
136	128115	>	128600		161		X		131 (100.0)	122 (38.1)	131 (66.1)	169 (62.7)	-	AOW41495 (62.7)	163 (63.6)	165 (62.7)	-		
137	128633	<	131206	*enhancin-2*	857	X	X	X	132 (99.5)	-	140 (43.45)	170 (42.2)	140 (43.45)	AOW41496 (42.2)	164 (42.2)	166 (42.3)	-		
138	131352	>	131798		146	X			133 (96.6)	-	-	171 (34.0)	141 (28.8)	AOW41497 (33.3)	165 (37.7)	167 (39.6)	-	x	
139	131855	>	132343	*chit-2c*	162	X	X	X	134 (99.4)	-	-	-	-	-	-	-	-		
hr8	133555	>	133603																

140	132617	>	133207	*Ac53*	196		X		135 (100.0)	127 (44.7)	134 (57.25)	173 (61.9)	143 (68.35)	AOW41500 (61.9)	169 (60.1)	171 (60.9)	134 (47.0)		ac53
141	133188	<	134441		417		X	X	136 (99.76)	128 (32.5)	135 (57.2)	174 (60.9)	144 (57.0)	AOW41501 (60.9)	170 (56.6)	172 (56.3)	-		
142	134629	>	134847	*lef-10*	72			X	137 (100.0)	-	-	176 (72.9)	146 (76.1)	AOW41503 (72.9)	172 (71.4)	174 (71.4)	-		ac53a
143	134448	<	134651		67		X	X	138 (100.0)	129 (36.4)	136 (62.7)	175 (76.1)	145 (73.1)	AOW41502 (76.1)	171 (68.7)	173 (68.7)	-	x	
144	134720	>	135694	*vp1054*	324				139 (99.1)	130 (49.8)	137 (75.8)	177 (75.5)	147 (72.4)	AOW41504 (75.5)	173 (74.2)	175 (74.5)	138 (44.9)		ac54
145	135732	>	135965		77				140 (100.0)	131 (57.6)	138 (66.7)	178 (68.3)	148 (67.8)	AOW41505 (68.3)	174 (61.4)	176 (61.4)	-	x	
146	135958	>	136365		106				141 (100.0)	-	140 (38.0)	179 (35.6)	149 (45.2)	AOW41506 (35.6)	175 (36.1)	177 (37.0)	-	x	
147	136399	>	137256	fgf-3	285		X		142 (98.6)	133 (40.1)	141 (45.8)	180 (44.8)	150 (58.5)	AOW41507 (44.8)	176 (45.6)	178 (46.4)	140 (19.5)		
148	137273	>	137902		209	X	X		143a (97.1)	-	142 (25.3)	181 (28.6)	151 (27.3)	AOW41508 (28.6)	177 (28.4)	179 (28.4)	-	x	
149	137965	>	138585		190	X	X		143b (99.0)	-	142 (29.5)	181 (28.3)	151 (29.6)	AOW41508 (28.3)	177 (29.8)	179 (29.8)	-	x	
150	138589	>	139485	me53	298			X	144 (99.3)	134 (40.75)	143 (63.7)	182 (66.7)	152 (63.7)	AOW41509 (66.7)	178 (64.9)	180 (64.9)	143 (34.7)		ac139
151	139487	>	139786		99	X	X	X	145 (99.0)	-	144 (59.6)	183 (63.6)	153 (67.35)	AOW41510 (63.6)	179 (62.6)	181 (61.6)	-	x	
