# Supplementary material for: Genomic analysis of an Argentinean isolate of Spodoptera frugiperda granulovirus reveals that various baculoviruses code for Lef-7 proteins with three F-box domains
Source: PLoS One. 2018 Aug 22;13(8):e0202598. doi: 10.1371/journal.pone.0202598 (PMC6105029; doi:10.1371/journal.pone.0202598)
Supplement: S2 Table — (PDF) [file pone.0202598.s002.pdf]

S2 Table. Accession number list of genomes used in Fig 2.

Baculovirus	Acc. number
Adoxophyes orana GV	NC_005038
Adoxophyes orana GV—Strain Miyazaki	KM226332
Agrotis segetum GV	NC_005839
Agrotis segetum GV—Strain L1	KC994902
Agrotis segetum GV—Strain DA	KR584663
Choristoneura occidentalis GV	NC_008168
Clostera anacoreta GV—Isolate HBHN	NC_015398
Clostera anastomosis GV—Strain Henan	NC_022646
Cnaphalocrocis medinalis GV – Strain Enpig	NC_029304
Cnaphalocrocis medinalis GV	KP658210
Cryptophlebia leucotreta GV	NC_005068
Cydia pomonella GV	NC_002816
Cydia pomonella GV—Isolate E2	KM217577
Cydia pomonella GV—Isolate S	KM217573
Cydia pomonella GV—Isolate I12	KM217576
Cydia pomonella GV—Isolate M	KM217575
Cydia pomonella GV—Isolate I07	KM217574
Diatraea saccharalis GV—Isolate Parana-2009	NC_028491
Epinotia aporema GV	NC_018875
Erinnyis ello GV—Strain BrS86	NC_025257
Helicoverpa armigera GV	NC_010240
Mythimna unipuncta GV – Isolate #8	NC_033780
Mocis sp GV	NC_029996
Phthorimaea operculella GV	NC_004062
Phthorimaea operculella GV – Strain SA	KU666536
Pieris rapae GV	NC_013797
Pieris rapae GV—Strain-E3	GU111736
Pieris rapae GV—South Korea	JX968491
Plodia interpunctella GV – Strain Cambridge	NC_032255
Plutella xylostella GV	NC_002593
Plutella xylostella GV SA	KU666537
Plutella xylostella GV K	KU529792
Plutella xylostella GV M	KU529793
Plutella xylostella GV T	KU529794
Plutella xylostella GV C	KU52979
Pseudaletia unipuncta GV—Strain Hawaiian	NC_013772
Spodoptera frugiperda GV—Isolate VG008	NC_026511
Spodoptera litura GV—Strain K1	NC_009503
Trichoplusia ni GV – Isolate LBIV-12	KU752557
Xestia c-nigrum GV	NC_002331
