# Supplementary material for: Genomic analysis of an Argentinean isolate of Spodoptera frugiperda granulovirus reveals that various baculoviruses code for Lef-7 proteins with three F-box domains
Source: PLoS One. 2018 Aug 22;13(8):e0202598. doi: 10.1371/journal.pone.0202598 (PMC6105029; doi:10.1371/journal.pone.0202598)
Supplement: S3 Table — (PDF) [file pone.0202598.s003.pdf]

S3 Table. Uniprot IDs of lef-7 homologs used in Fig 3.

**Acronym in Fig. 3**	**Virus name (isolate or strain)**	**Uniprot ID**
ORF51_SfGV-ARG	Spodoptera frugiperda granulovirus (ARG)	
ORF049_SfGV-VG008	Spodoptera frugiperda granulovirus (VG008)	A0A0C5B323
ORF084_SfGV-VG008	Spodoptera frugiperda granulovirus (VG008)	A0A0C5B350
PsunGV	Pseudaletia unipunta granulovirus (Hawaiin)	B6S703
TniGV	Trichoplusia ni granulovirus (LBIV-12)	A0A1D8QLD3
XcenGV	Xestia c-nigrum granulovirus	Q9PYR6
HearGV	Helicoverpa armigera granulovirus	A9YMX4
SeMNPV_HT-SeG25	Spodoptera exigua multiple nucleopolyhedrovirus (HT-SeG25)	W0UYT7
SeMNPV_VT-SeAl1	Spodoptera exigua multiple nucleopolyhedrovirus (VT-SeAl1)	W0UZS9
SfMNPV_3AP2	Spodoptera frugiperda multiple nucleopolyhedrovirus (3AP2)	A1YJ11
SfMNPV_Nic	Spodoptera frugiperda multiple nucleopolyhedrovirus (Nicaraguan)	E9L613
SfMNPV_Col	Spodoptera frugiperda multiple nucleopolyhedrovirus (Colombian)	A0A0R5RHP7
SfMNPV_19	Spodoptera frugiperda multiple nucleopolyhedrovirus (19)	B2KWV4
AgseNPV	Agrotis segetum nucleopolyhedrovirus	Q287Q1
