# Supplementary material for: Genomic analysis of an Argentinean isolate of Spodoptera frugiperda granulovirus reveals that various baculoviruses code for Lef-7 proteins with three F-box domains
Source: PLoS One. 2018 Aug 22;13(8):e0202598. doi: 10.1371/journal.pone.0202598 (PMC6105029; doi:10.1371/journal.pone.0202598)
Supplement: S1 Appendix — (PDF) [file pone.0202598.s004.pdf]

S1 Appendix

RT-PCR of 5 SfGV ORFs using mRNAs from *S. frugiperda* dead larvae infected with SfGV as a template.

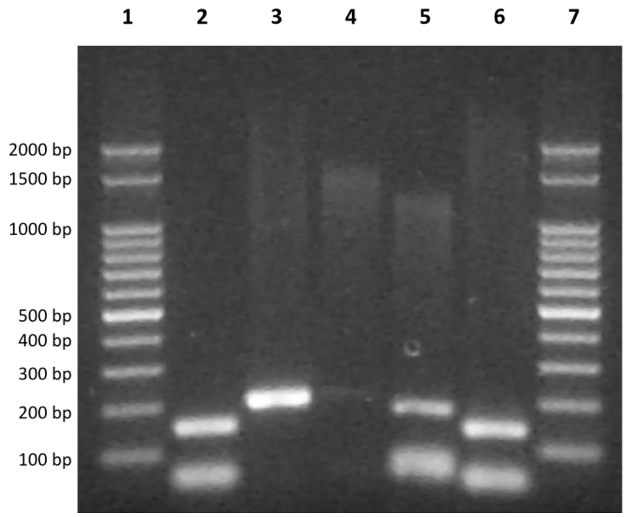

Lanes:

1: ladder 100 bp
2: Granulin
3: ORF031
4: ORF056
5: ORF057
6: ORF122
7: ladder 100 bp

Primers:

Granulin	sequence	Amplicon size (nt)
FW	CGCGAGTCTCAGTACGATCCG	153
RV	CCAGTTGCACACCAGTTTCATGG	

ORF031	sequence	Amplicon size (nt)
FW	CGAACGAGAGGTGGACTAC	207
RV	GCAGACACAATGAGTCTAGAATG	

ORF056	sequence	Amplicon size (nt)
FW	TTCATCAACTCACCCCGCTG	214
RV	TCCTTCGCCTCTTCCAGATAC	

ORF057	sequence	Amplicon size (nt)
FW	TGGTACCTTCGTTTGCACTGC	178
RV	ACAACCCGTACACGTCGAAC	

ORF122	sequence	Amplicon size (nt)
FW	ATCAGCGAGCTCATTTCACC	140
RV	GGTGTACTGTCTTACTTGCC	
