# Supplementary material for: Genomic analysis of an Argentinean isolate of Spodoptera frugiperda granulovirus reveals that various baculoviruses code for Lef-7 proteins with three F-box domains
Source: PLoS One. 2018 Aug 22;13(8):e0202598. doi: 10.1371/journal.pone.0202598 (PMC6105029; doi:10.1371/journal.pone.0202598)
Supplement: S2 Appendix — (PDF) [file pone.0202598.s005.pdf]

S2 Appendix. ORF 066

A. Localization of ORF066 in AfGV ARG genome

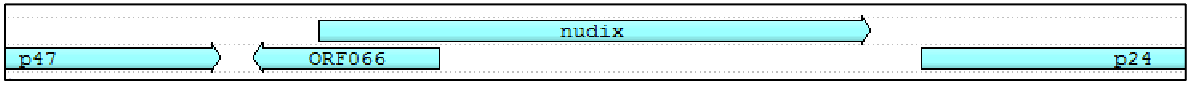

B. Table. ORF066 homolog peptides found in alpha- and betabaculovirus

Genome	Accession	Strain/isolate	Length (aa)	ORF location	Overlapped with ORF n° (nudix)	promotor motifs (300 nt upstream ATG)
Spodoptera frugiperda granulovirus		ARG	74	59757 < 59981	67	4 x CAKT
Spodoptera frugiperda granulovirus	NC_026511	VG008	75	59914 < 60138	64	4 x CAKT
Pseudaletia unipuncta granulovirus	NC_013772	Hawaiin	79	77975 < 78211	82	TATAA + 5 x CAKT
Spodoptera litura granulovirus	NC_009503	SlGV-K1	29	45555 < 45641	66	TATATAT + 2x CAGT, TATAT
Xestia c-nigrum granulovirus	NC_002331	-	47	75793 < 75933	79	6 x CAKT
Trichoplusia ni granulovirus	KU752557		79	75864 < 76100	AOW14412.1	TATAA + 5 x CAKT
Helicoverpa armigera granulovirus	NC_010240	-	100	72801 < 73106	77	TATAA + 4 x CAKT
Mocis sp. granulovirus	NC_029996	Southern Brazil	98	55772 < 56065	64	TATAAA, TTAAG
Mythimna unipuncta granulovirus	NC_033780	MyunGV#8	92	65223 < 65498	71	CATT
Agrotis segetum granulovirus	NC_005839	-	56	62636 < 62803	61	not analized
Plutella xylostella granulovirus	NC_002593	K1	-	-	-	
Adoxophyes orana granulovirus	NC_005038	-	23	42875 < 42943	59	not analized
Choristoneura occidentalis granulovirus	NC_008168	-	-	-	-	not analized
Clostera anachoreta granulovirus	NC_015398	ClanGV-HBHN	24	44519 < 44590	56	not analized
Clostera anastomosis granulovirus Henan	NC_022646	CaLGV-Henan	24	44397 < 44468	59	not analized
Clostera anastomosis granulovirus B	KR091910		45	46057 < 46191	55	not analized
Cnaphalocrocis medinalis granulovirus	NC_029304	Enping	64	46610 < 46801	51	not analized
Cryptophlebia leucotreta granulovirus	NC_005068	CV3	-	-	-	
Cydia pomonella granulovirus	NC_002816	Mexican 1	43	55766 < 55894	69	not analized
Diatraea saccharalis granulovirus	NC_028491	Parana-2009	28	41724 < 41807	58	not analized
Epinotia aporema granulovirus	NC_018875	-	23	60980 < 61048	65	not analized
Erinnyis ello granulovirus	NC_025257	S86	85	43160 < 43414	62	not analized
Phthorimaea operculella granulovirus	NC_004062	-	64	53195 < 53386	62	not analized
Pieris rapae granulovirus	NC_013797	Wuhan	49	49314 < 49460	57	not analized
Plodia interpunctella granulovirus	NC_032255	Cambridge	-	-	-	not analized
Autographa californica nucleopolyhedrovirus	L22858	C6	48	30881 > 31027	38	not analized
Plutella xylostella multiple nucleopolyhedrovirus	DQ457003	CL3	48	32151 > 32297	38	not analized
Bombyx mori nucleopolyhedrovirus	L33180	T3	48	26969 > 27115	29	not analized
Maruca vitrata MNPV	EF125867		48	23715 > 23861	27	not analized
Thysanoplusia orichalcea NPV	JX467702	p2	48	30474 > 30620	36	not analized
Dendrolimus kikuchii nucleopolyhedrovirus	JX193905	strain YN	164	101073 > 101567	113, 112	not analized
Lonomia obliqua multiple nucleopolyhedrovirus	KP763670	SP/2000	32	96469 < 96567	106	not analized
Anticarsia gemmatalis nucleopolyhedrovirus	DQ813662		70	113157 > 113369	142	not analized
Condylorrhiza vestigialis MNPV	KJ631623		55	108155 > 108322	119	not analized
Epiphyas postvittana nucleopolyhedrovirus	AY043265		78	16214 < 16450	20	not analized
Philosamia cynthia ricini nucleopolyhedrovirus	JX404026		32	119493 > 110591	118	not analized
Antheraea pernyi nucleopolyhedrovirus	EF207986	L2	32	111870 > 111968	127	not analized
Hyphantria cunea nucleopolyhedrovirus	AP009046		16	117154 > 117201	129	not analized
Choristoneura occidentalis alphabaculovirus	KC961303			-		
Choristoneura rosaceana alphabaculovirus	KC961304			-		
Pseudoplusia includens SNPV	KJ631622	IE	83	27682 > 27933	31	not analized
Dasychira pudibunda nucleopolyhedrovirus	KP747440	L1	39	119693 > 119812	140	not analized
Trichoplusia ni single nucleopolyhedrovirus	DQ017380		97	26448 > 26741	28	not analized

Spodoptera exigua NPV	AF169823		27	115897 < 116070	118	not analized
Agrotis segetum B NPV	KM102981	English	27	129445 < 129528	127	not analized
Mamestra configurata nucleopolyhedrovirus A	AF539999	90/4	22	136153 < 136221	148	not analized
Helicoverpa armigera multiple nucleopolyhedrovirus	EU730893		19	136691 < 136750	141	not analized
Peridroma alphabaculovirus	KM009991	GR_167	27	131019 < 131102	119	not analized

C. Multiple alignment of SfGV ARG ORF066 and its homologs of closely related betabaculoviruses

```
HearGV      --------MCLLYFKFLLTCSLYDLFACKITALSSSVMSNSPECLPFLTIVRHSYTTLPYRINTHPIIYQPLSQHNLTIIKMPLNLHKNYHHFSHLIATTESQSCVDKT
XcenGV      -------------------------------------MSNSPECLPFLTIVRHSYTTLPYRINTHPIIYQPLSQNNLTIIKTL--------------------------
Mocis sp GV --------MCLLYLRFLFTCSLYDLFACKITALSSFVISNNPECLPFLTILRHSCTTLPYRTTHNALYTKRYLSINRSHIVKHNNVNNFRKKLPKISTSHHHQIQ----
SfGV-ARG    MLSFSTNGMCLLYFRFLFTCSLYDLFACKMTALSSSVISKSPECLPFLTIARHSYAALPYKRPRNPLYTHVIYY-----------------------------------
SfGV-VG008  MLSFSTNGMCLLYFRFLFTCSLYDLFACKMTALSSSVISKSPECLPFLTIARHSYAALPYKRPRNPLYTHVIYY-----------------------------------
PsunGV      --------MCLLYFRFLFTCSLYDLFACKITALSSSVMSNSPECLPFLTIVRHSYTTLPYRINTQPIIYLTLSLHNQSTQSLIIIK-----------------------
TniGV       --------MCLLYFRFLFTCSLYDLFACKITALSSSVMSNSPECLPFLTIVRHSYTTLPYRINTQPIIYLTLSLHNQSTQSLIIIK-----------------------
SpliGV      ----------------------------------------MTTFLNHLDKVFNNNTRLPYFILTSLDS-----------------------------------------
```
