# Supplementary material for: Genomic analysis of an Argentinean isolate of Spodoptera frugiperda granulovirus reveals that various baculoviruses code for Lef-7 proteins with three F-box domains
Source: PLoS One. 2018 Aug 22;13(8):e0202598. doi: 10.1371/journal.pone.0202598 (PMC6105029; doi:10.1371/journal.pone.0202598)
Supplement: S3 Appendix — (PDF) [file pone.0202598.s006.pdf]

S3 Appendix. ClbiNPV and ApciNPV photolyase genes.

Clanis bilineata NPV and Apocheima cinerarium *photolyase* annotation and their putative frameshift after BlastX analysis.

A. Clanis bilineata nucleopolyhedrovirus (GenBank: DQ504428):

ORFs annotated as DNA photolyase 1 and DNA photolyase 2 are coded in reading frames -1 and -3, respectively. Blastx search revealed a complete photolyase which starts in frame with DNA photolyase 2 (RF -1) and continues in frame with DNA photolyase 2 (RF -3).

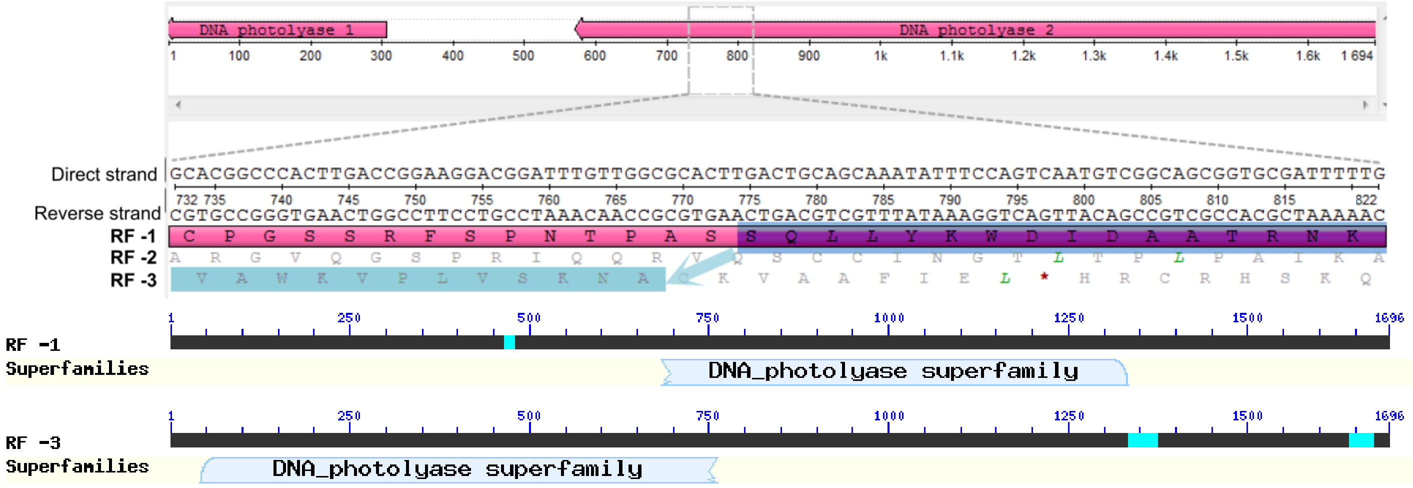

Translation after putative frameshift:

MSSSRSVATSTLTQSSTSCVDKKVKRRSRDHISSSVEKKFKTDSKRKSISKSSFAADDHNDSENVSQDHDDGHMRLLNEFRANR
SLNHDIKFDPRRVRILKLGRSRTAVVNDDTLNGDVDGGVVYWMSRDSRVQDNWAMIYAQRLAIERQVPLHVCFCLTSSFLNASL
RQFDFLLEGLKSVQRECHELNIGFHVLDGSGDLTLNDWVQTNNISAVVCDFNPLRVVRDWVATVKSQLAPHVFFAQVDAHNIVP
CWQASLKQEVYASTFRPKVLNQLDRFLTEFPSVIVHPYGKNRTAADIDWKYLLQSXXANKSVLPVKWAVPGYDHAVKSLYIFIQ
DNLSKYASLRNNPTTTVISNLSPWFHFGQISVQRVVLKILSLKPSHPDSVDRYIDEVVVRRELADNFCFYNDRYDCVEGAPQWA
QETIEQHKYDHRSYIYNLTELASCQTHDELWNSAQAQLRCEGKMHGYMRMYWAKKIMEWTPSADMALGYAIFLNDHYSVDGRDP
NNYVGCMWSICGVHDRPFKERKIFGRIRYMNYAGCKRKFNVDLYIAKYKIKNSQQECVIV*

B. Apocheima cinerarium nucleopolyhedrovirus (NC_018504)

ORFs annotated as DNA photolyase 1 and DNA photolyase 2 are coded in reading frames -1 and -2, respectively. Blastx search revealed a complete photolyase which starts in frame with DNA photolyase 2 (RF -1) and continues in frame with DNA photolyase 2 (RF -2).

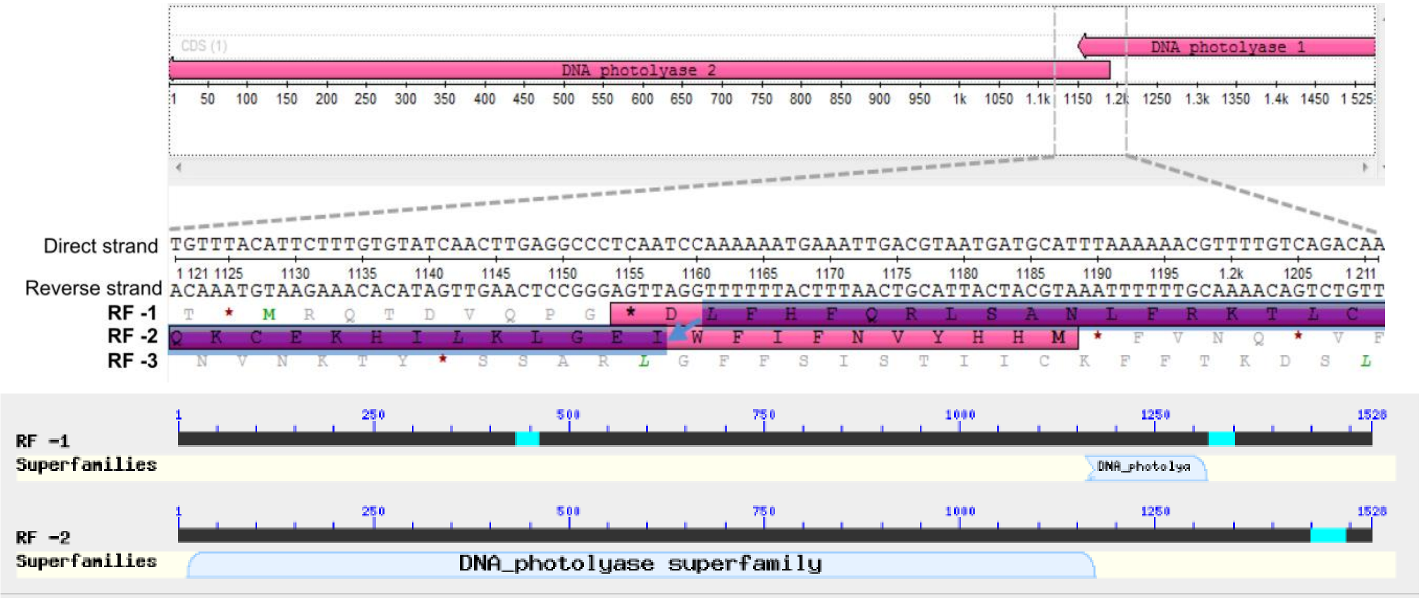

Translation after putative frameshift:

MSKSLSYLNFPPKRVKLNSSVETAEELMKKFHQSRIENSMNKCDPRRIRVLSSLCQSVNQNANNNGRNGGIVYWMSRDSRVQ
DNWAMIYAQELANAQNLPLYVVFCLTKRFLNASLRQFHFLIEGLKLIHKECKQLNITFVVLNGSGDETLVDWVKKYNIAGIV
CDFNPLRIVRRWTARVKTQLPSDVYFAQIDAHNIVPCWVASQKQEINARTMRNKLKTNMKSFLKPFPLVMKHSIDSKARIDP
ATCDEIDWKKLLESRDADKNIEPVTWAQAGYNNACVALAKFIDDNLWHYKETRNDPNADSQSNMSPWYHFGQISVQRVVWYL
IVAKMQHFESNVETYIEECFVRRELADNFCYYNINYDRFEGAPDWAKETLSLHANEGREYCYNRRELENSETHDILWNAAQT
QLKTQGKMHGYMRIYWAKKILEWSISPTVALINAIYFNDKYSLDGRDPNGYAGCMWSICGTHDRAWMERPIYGKIRHMNFEG
CKRKFNIDCYINKNK
