# Supplementary material for: Genomic analysis of an Argentinean isolate of Spodoptera frugiperda granulovirus reveals that various baculoviruses code for Lef-7 proteins with three F-box domains
Source: PLoS One. 2018 Aug 22;13(8):e0202598. doi: 10.1371/journal.pone.0202598 (PMC6105029; doi:10.1371/journal.pone.0202598)
Supplement: S1 Fig — (PDF) [file pone.0202598.s007.pdf]

S1 Fig. Relative similarity plot: SfGV-ARG vs. SfGV VG008

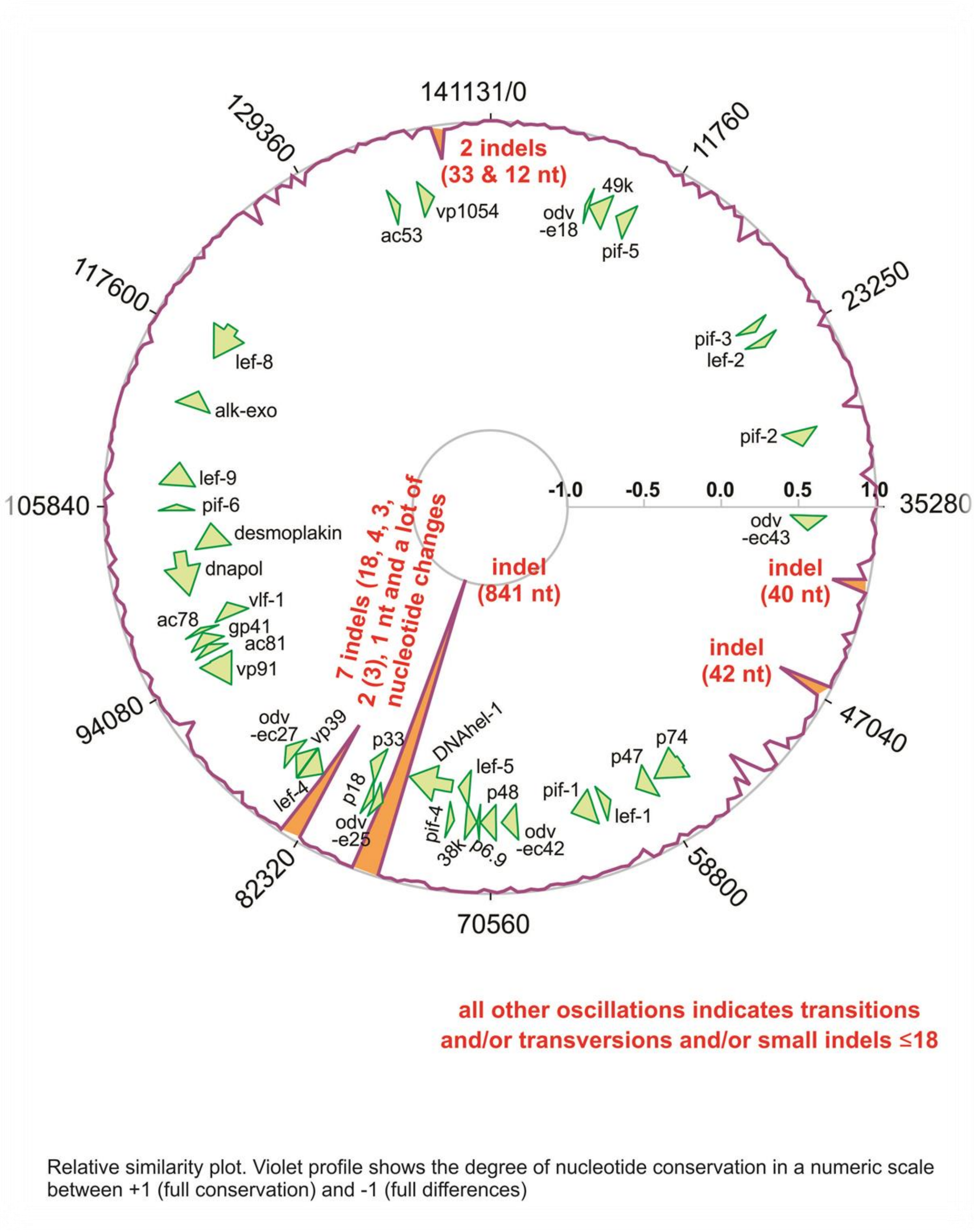

Relative similarity plot between SfGV-VG008 and SfGV-Arg. Violet profile shows the degree of nucleotide conservation in a numeric scale between +1 (full conservation) and -1 (full difference). Inner and outer gray circles are references for both extreme values. Nucleotide positions correspond to the pairwise alignment. Most of the changes are transitions, transversions or small indels (≤18). The biggest differences are indicated (shaded in orange and numbered): (1) intergenic region, (2) deletion in frame inside bro-b, (3) lef-7 deletion, (4) changes in frame inside bro-e, (5) deletions in frame inside fgf-3. Core genes are indicated in order to shown that the biggest differences do not affect these essential genomic information.
