# Supplementary material for: Genomic analysis of an Argentinean isolate of Spodoptera frugiperda granulovirus reveals that various baculoviruses code for Lef-7 proteins with three F-box domains
Source: PLoS One. 2018 Aug 22;13(8):e0202598. doi: 10.1371/journal.pone.0202598 (PMC6105029; doi:10.1371/journal.pone.0202598)
Supplement: S2 Fig — (PDF) [file pone.0202598.s008.pdf]

S2 Fig. Multiple alignment of ORF020 homologs

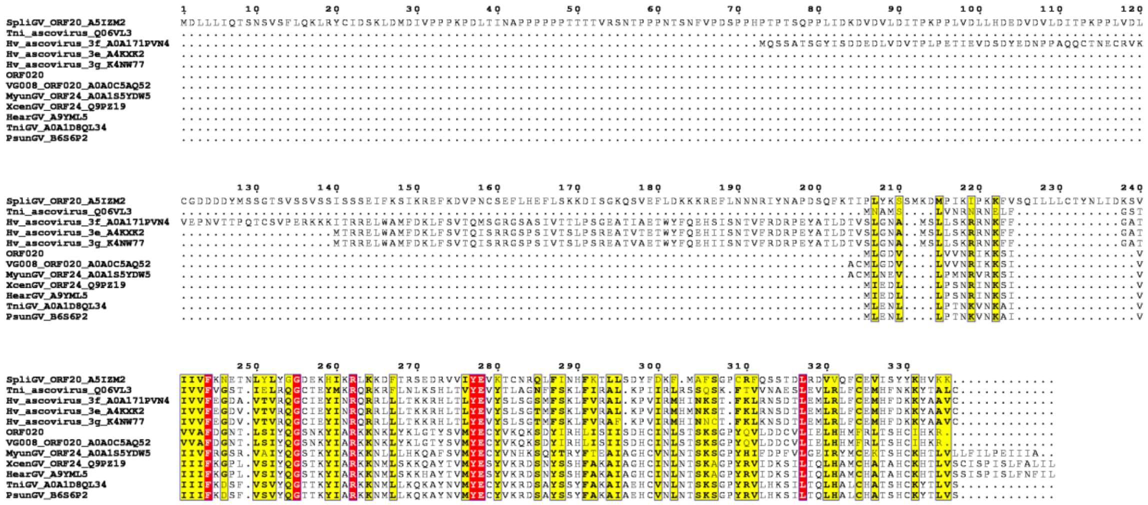

Multiple alignment (MUSCLE) of SfGV ARG ORF020 with its homologs in SfGV VG008, SpliGV, MyunGV, XcenGV, HearGV, PsunGV, TniGV, HvAV and TniAV. Uniprot IDs are indicated.
