# Supplementary material for: Genomic analysis of an Argentinean isolate of Spodoptera frugiperda granulovirus reveals that various baculoviruses code for Lef-7 proteins with three F-box domains
Source: PLoS One. 2018 Aug 22;13(8):e0202598. doi: 10.1371/journal.pone.0202598 (PMC6105029; doi:10.1371/journal.pone.0202598)
Supplement: S4 Fig — (PDF) [file pone.0202598.s010.pdf]

S4 Fig. Multiple alignment of ORF058, ORF133 and their homologs.

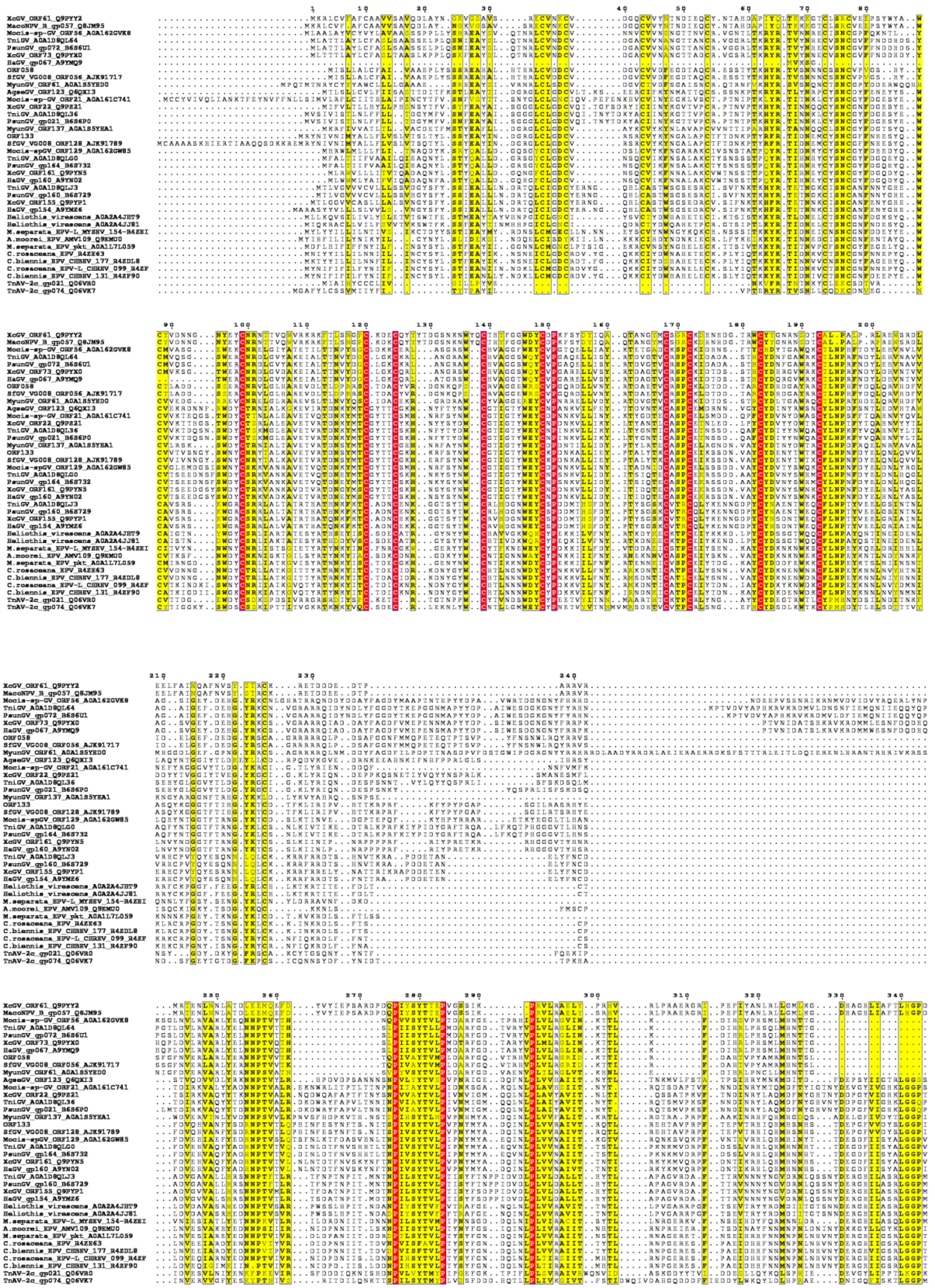

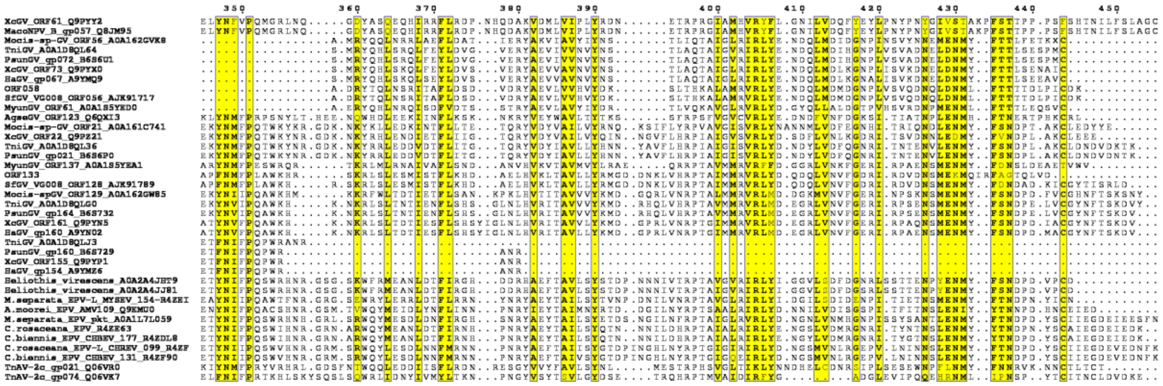

Multiple alignment of ORFs 058 and 133 and its homologs in baculovirus, enotomopoxvirus and ascovirus. Uniprot IDs are indicated. pkt: *parasitoid killer toxin*
