# Supplementary material for: Genomic analysis of an Argentinean isolate of Spodoptera frugiperda granulovirus reveals that various baculoviruses code for Lef-7 proteins with three F-box domains
Source: PLoS One. 2018 Aug 22;13(8):e0202598. doi: 10.1371/journal.pone.0202598 (PMC6105029; doi:10.1371/journal.pone.0202598)
Supplement: S5 Fig — (PDF) [file pone.0202598.s011.pdf]

S5 Fig. Multiple alignment of ORF060 homologs.

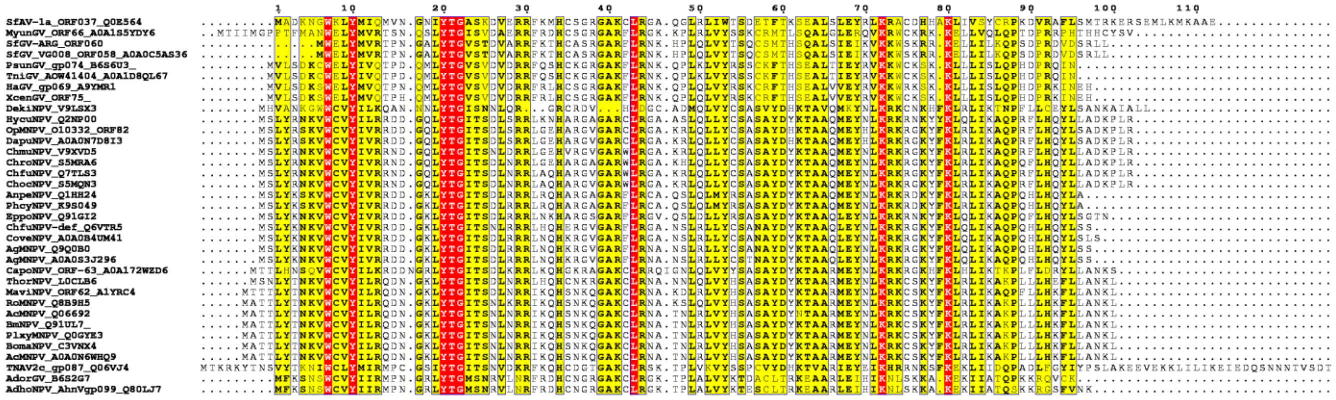

Multiple alignment (MUSCLE) of ORF060 and its homologs in baculovirus and ascovirus. Uniprot IDs are indicated.
